# Synthesis, Antitumor and Antibacterial Studies of New Shortened Analogues of (KLAKLAK)_2_-NH_2_ and Their Conjugates Containing Unnatural Amino Acids

**DOI:** 10.3390/molecules26040898

**Published:** 2021-02-08

**Authors:** Sirine Jaber, Ivan Iliev, Tsvetelina Angelova, Veronica Nemska, Inna Sulikovska, Emilia Naydenova, Nelly Georgieva, Ivan Givechev, Ivo Grabchev, Dancho Danalev

**Affiliations:** 1 University of Chemical Technology and Metallurgy, 8 Kliment Ohridski blvd., 1756 Sofia, Bulgaria; Jaber-Sirine@hotmail.com (S.J.); tsvetelina_angelova@abv.bg (T.A.); vnemska@uctm.edu (V.N.); neli@uctm.edu (N.G.); ivangivechev@gmail.com (I.G.); 2 Institute of Experimental Morphology, Pathology and Anthropology with Museum, Bulgarian Academy of Sciences, Acad. G. Bonchev str., bl. 25, 1113 Sofia, Bulgaria; taparsky@abv.bg (I.I.); inna_sulikovska@ukr.net (I.S.); 3 Testing Center Global Test Ltd., 31 Krushovski vrah Street, 1618 Sofia, Bulgaria; 4 Department of Chemistry and Biochemistry, Physiology and Pathophysiology, Sofia University “St. Kliment Ohridski”, 1504 Sofia, Bulgaria; i.grabchev@chem.uni-sofia.bg

**Keywords:** (KLAKLAK)_2_-NH_2_, anticancer peptides, 1,8-naphthalimide, caffeic acid, unnatural amino acids, antimicrobial activity, anticancer properties

## Abstract

(1) Background: (KLAKLAK)_2_ is a representative of the antimicrobial peptide group which also shows good anticancer properties. (2) Methods: Herein, we report synthesis using SPPS and characterization by HPLC/MS of a series of shortened analogues of (KLAKLAK)_2_. They contain single sequence KLAKLAK as C-terminal amides. In addition, substitution of some natural amino acids with unnatural β-Ala and nor-Leu is realized. In addition, these structures are conjugated with second pharmacophore with well proven anticancer properties 1,8-naphthalimide or caffeic acid. Cytotoxicity, antiproliferative effect and antimicrobial activity of newly synthesized structures were studied. (3) Results: The obtained experimental results reveal significant selective index for substances with common chemical structure KLβAKLβAK-NH_2_. The antibacterial properties of newly synthesized analogues at two different concentrations 10 μM and 20 μM, were tested against Gram-negative microorganisms *Escherichia coli K12 407*. Only two of the studied compounds KLAKLAK-NH_2_ and the one conjugated with second pharmacophore 1,8-naphthalimide and unnatural amino acid nor-Leu showed moderate activity against tested strains at concentration of 20 μM. (4) Conclusions: The obtained results reveal that the introducing of 1,8-naphthalimideGly- and Caf- increase the cytotoxicity and antiproliferative activity of the peptides but not their selectivity. Only two compounds KLAKLAK-NH_2_ and 1,8-naphthalimideGKnLAKnLAK-NH_2_ show moderate activity against *Escherichia coli K12* at low concentration of 20 μM.

## 1. Introduction

Most antimicrobial peptides contain 10 to 50 amino acids and are cationic with an amphipathic structure [1]. (KLAKLAK)_2_ is a representative of this group of peptides, whose primary structure consists of 14 amino acids [2,3]. Theoretically the selectivity and mechanism of action of antimicrobial and anticancer peptides are similar. There are experimental data that these properties depend on availability of total negative charge of bacterial membrane and the tumor surface, due to high content of anionic molecules there, such as glycoconjugates, heparin sulfate, etc. [1,4]. On the other hand the normal mammalian cells contained many zwitterion structures in their phospholipid layer like phosphatidylethanolamine, phosphatidylcholine, etc. which form a neutral total charge, making these cells less attractive for cationic antimicrobial peptides [5]. As a result, many peptides with proven antimicrobial activities have been tested and they showed anticancer effect [6,7,8]. Due to all mentioned above, antimicrobial peptides are of a large interest for scientific groups as possible alternative as anticancer compounds. Antimicrobial peptide (KLAKLAK)_2_ is one of those peptides which shows antitumor properties, as on internalization it causes mitochondrial swelling and destruction of the mitochondrial membrane leading to apoptosis [9]. There are data in literature concerning different (KLAKLAK)_2_ analogues and investigations on their anticancer potential [10]. Javadpour et al. described synthesis of peptides with the sequences:  (KLAKKLA)n, (KLAKLAK)n (where *n* = 1, 2, 3), (KALKALK)_3_, (KLGKKLG)_n_, and (KAAKKAA)_n_ (where *n* = 2, 3) as the C-terminal amides. They realized several tests for cytotoxicity of those compounds and concluded that the peptides were much less lytic toward human erythrocytes than 3T3 cells at concentrations lower than 22 μM [2].

Introducing of unnatural amino acids in peptide structure regularly leads to changing, often increasing of main activity of the peptide. Thus many peptides with improved general activity are created such as anticancer analogues of somatostatin introduced in a medical practice octreotide and lanreotide [11,12,13,14], anticoagulant peptides [15,16,17,18], antiviral peptides [19,20,21], etc. Herein, we report the synthesis as well as cytotoxicity and antitumor studies of new shortened analogues of antimicrobial peptide (KLAKLAK)_2_-NH_2_ containing unnatural amino acids β-Ala and nor-Leu as well as their conjugates containing 1,8-naphthalimide and caffeic acid.

## 2. Results

A series of shortened analogues of (KLAKLAK)_2_ as C-terminal amides with general structure Lys-X-Y-Lys-X-Y-Lys-NH_2_, where X is Leu or nor-Leu (nL) and Y is Ala or β-Ala (β-A) were synthesized. In addition, their conjugates with general structure Z-Lys-X-Y-Lys-X-Y-Lys-NH_2_, where X and Y are amino acids already mentioned above and Z is 1,8-naphthalimide-Gly or caffeic acid (Caf), were also obtained. (KLAKLAK)_2_-NH_2_ as a standard for further biological tests was also synthesized. All compounds were synthesized by SPPS, Fmoc/OtBu strategy. C-terminal amides were obtained by means of Rink-amide MBHA resin as solid-phase carrier. All condensation steps were realized with 3-[Bis(dimethylamino)methyliumyl]-3H-benzotriazol-1-oxide hexafluorophosphate (HBTU) or N,N′-Diisopropylcarbodiimide (DIC) as condensation reagents. Analytical data for newly synthesized peptides (Supplementary Materials) are summarized in Table 1.

### 2.1. Cytotoxicity

**Figure 1:**
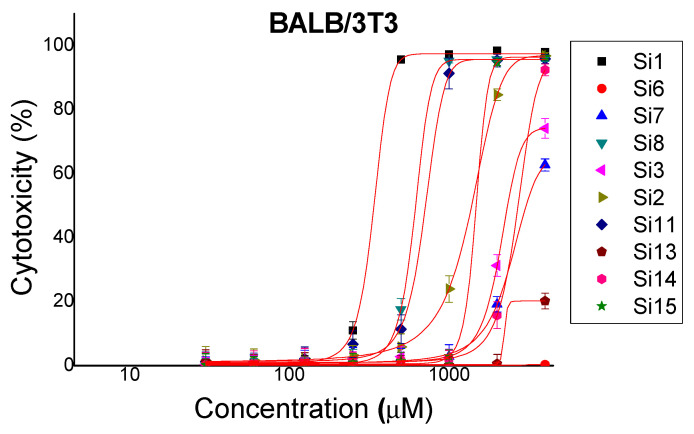
Cytotoxic effects of newly synthesized compounds. Dose-response curves for cytotoxicity assessment in BALB/3T3 cells.

The newly synthesized compounds were studied for cytotoxicity by standard method (3T3 NRU-test). The cells were incubated with the test substances at a concentration of 30 to 4000 μM for 24 h. The cytotoxicity expressed, in % relative to the negative control was determined. Dose-response dependence was observed for all substances. The obtained results are shown on Figure 1.

The lowest toxicity was observed in **Si6** where no significant difference was observed compared to the negative control (untreated cells) and **Si13** where 20% cytotoxicity was observed at the highest concentration studied (4 mM). At a concentration of 250 μM, no cytotoxic effect was observed on the test substances. Based on dose–response curves, IC_50_ values were calculated by nonlinear regression analysis Table 2. According to IC_50_ values, the most toxic substance was **Si1** with IC_50_ = 365.3 ± 4.076 μM followed by **Si8** with IC_50_ = 710.3 ± 11.91 μM and **Si11** with IC_50_ = 742.5 ± 18.49 μM. IC_50_ values for the other test substances are above 1000 μM, which is indicative of a low level of toxicity.

### 2.2. Antiproliferative Activity

**Figure 2:**
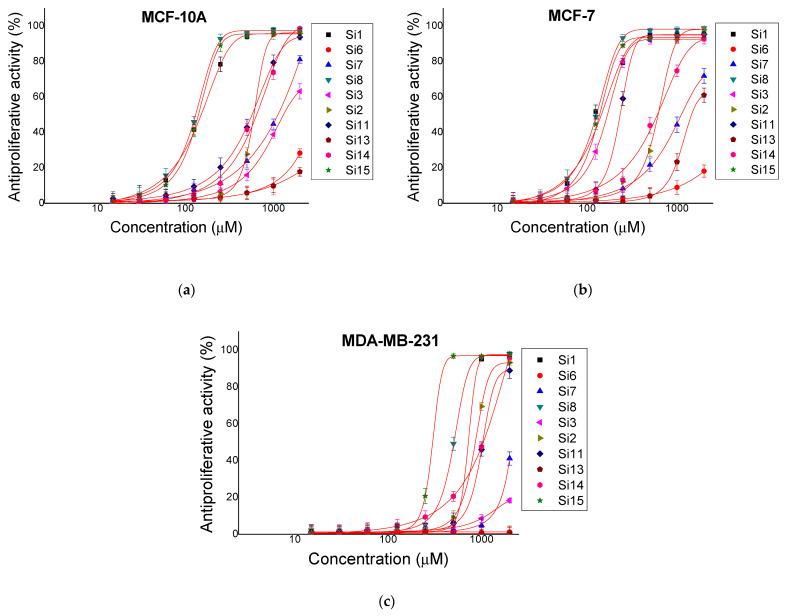
Antiproliferative activity of newly synthesized compounds. Dose-response curves assessment in (**a**) Breast, non-tumorigenic epithelial cells (MCF-10A), (**b**) mammary gland type A adenocarcinoma (MCF-7) and (**c**) triple-negative breast cancer (MDA-MB-231).

The compounds were studied for antiproliferative activity by standard MTT dye reduction assay. Cell cultures from different cell line types (MCF-10A, MCF-7 and MDA-MB-231) were incubated with the test substances at a concentration of 15 to 2000 μM for 72 h. The antiproliferative activity expressed in % relative to the negative control was determined. The obtained results are shown on Figure 2. The IC_50_ values of the mean were calculated and presented in Table 2. MCF-10A is a reliable model for normal human mammary epithelial cells, which serves as a control in experiments to determine antitumor activity. The IC_50_ values, found in MCF-10A, are used to calculate a selective index (SI), which assesses the potential of a substance to be used as an antitumor agent. We used the following formula to calculate the selective index SI = IC_50_ of MCF-10A / IC_50_ of tumor cells. The highest selective index with respect to MCF-7 is shown by the substances: **Si3** (SI = 8.35), **Si11** (SI = 2.62) and **Si1** (SI = 1.24). The calculated selective index in the positive control (Cisplatin) is 2.36. With respect to MDA-MB-231, SI < 1 was observed for any of the test substances. The widely used in clinical practice cytostatic Cisplatin (positive control) showed SI = 25.

### 2.3. Antibacterial Activity

**Figure 3:**
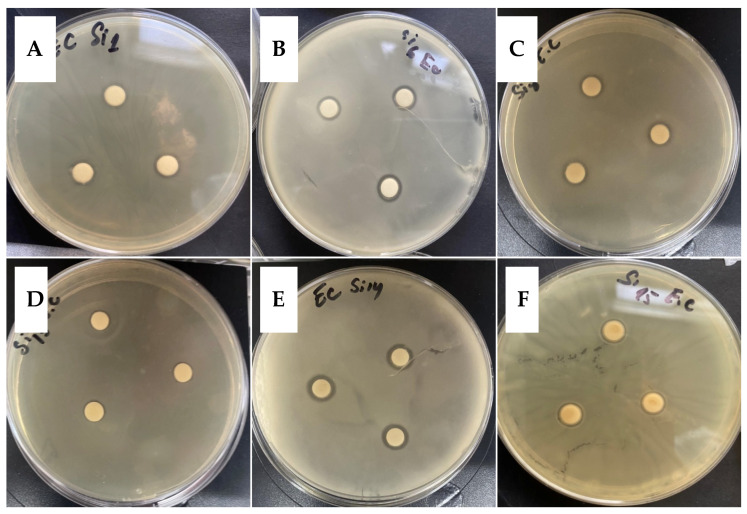
Antibacterial activity measured by the agar disk-diffusion method against *E. coli K12 407* as test microorganisms for: **Si1** (**A**), **Si6** (**B**), **Si8** (**C**), **Si13** (**D**), **Si14** (**E**), **Si15** (**F**).

Antibacterial properties of the obtained new peptides and their conjugates were tested against *E. coli* K12 407 strain. Antibacterial activity of tested compounds was proven through appear of free zone around the loaded disk-paper by means of agar-diffusion method (Figure 3).

The obtained results demonstrated clear zones free of microbial growth which means antibacterial effect around the samples **Si1**, **Si6**, **Si8** and **Si14** (Table 3). For the other compounds similar to **Si13** and **Si15** activity was found.

## 3. Discussion

One of the main problems in medicinal therapy in general is intracellular transport of biologically active substance. In addition, especially concerning cancer therapy, the problem of tumor targeting also arises. Recently “targeted therapy” is one of the promising alternatives of chemotherapeutics in the fight against increasing cancer illness. Peptides can be specifically designed according to the needs and currently they are largely used as delivery systems for different purposes [22,23,24,25]. Nowadays, peptides are often used as conjugates able to transport and deliver different therapeutic molecules to specific targets in the organism, the process is well known as vectorization [26,27]. Moreover, the peptide can be conjugated to a cytotoxic drug to deliver it to the cancer cells expressing the corresponding peptide receptor [28] or attracting it to the specific features of tumor cells.

The investigation of Javadpour et al. reports that the 7-mer analogues of (KLAKLAK)_2_ are devoid of tested biological activity [2]. Taking into account the fact that antimicrobial peptides have different mechanisms to penetrate cell membranes [29,30,31] we decided to conjugate 7-mer analogues of (KLAKLAK)_2_ with second pharmacophore in order to test vectorizing potential of this peptide according to different cell lines. In addition, antitumor activity of obtained hybrid molecules was tested. Caffeic acid is well known and widely distributed in different natural products, with many proven positive effects and properties such as antimicrobial activity [32], antioxidant properties [33] and especially anticancer activity [34,35,36,37,38,39]. Due to the high similarity between the cell membrane in prokaryotes and the outer membrane in mitochondria, it is logical to conclude that the target of antibacterial peptides, administered to mammalian cells, is the outer mitochondrial membrane. When the peptide (KLAKLAK)_2_-NH_2_ and its derivatives interact with the outer mitochondrial membrane, they disrupt its structure and functionality. As a result, membrane permeability is increased and cytochrome C, ROS and other substances that activate apoptosis are released from the mitochondria. In addition, there is significant data in literature about the anticancer properties of 1,8-naphtalimide and its derivatives [40,41,42,43,44] are already used in medicinal practice [45,46]. Moreover, 1,8-naphtalimide molecule in our bioconjugates contributes for fluorescent properties. They can be used in a further investigation to evaluate cell penetration ability and intracellular distribution of newly synthesized compounds.

The substitutions of the natural amino acids Ala and Leu with their unnatural analogues β-alanine and nor-Leu were made, taking into account several important facts, supported by many results in the scientific literature:

The replacement of natural with unnatural amino acids makes resulting peptides difficult to be recognized as substrates by the enzymes responsible for their hydrolysis in the body. This often leads to increased hydrolytic stability and half-life of the obtained compounds in human plasma, which is important for their candidature as potential medical drugs;

A number of authors prove that single substitutions of natural with non-natural amino acids lead to improvement of specific properties or biological activity of newly synthesized compounds [11,12,13,14,15,16,17,18,19,20,21,47,48];

Beta-amino analog of Ala was selected to be introduced into the primary structure of aim peptides because it will lead to obtaining of more rigid incapable of conformational freedom structures. Such kinds of structures are more stable of hydrolysis in acid or basic condition [49]. On the other hand a lot of authors change natural amino acid Leu with nor-Leu because the second one is simultaneously a structural analogue of Ile and Met. Most recently, Chan et al. show that this kind of substitution in short peptides results in obtaining of structures with a higher tendency of self-organization, which support the interaction with the phospholipid membranes of the bacterial cell and enhance the biological effect of the peptide itself or a molecule carried by it [47,48].

**Figure 4:**
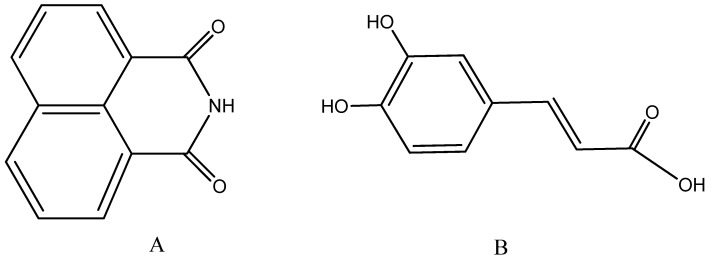
Chemical structures of second introduced in the molecule pharmacophore (**A**) naphtalimide and (**B**) caffeic acid.

All target peptides were synthesized using standard protocol of SPPS Fmoc/OtBu strategy on Rink-amide MBHA resin in order to assure final C-terminal amide, without any specific problems during the synthesis. If the step of condensation of some amino acid needed to be repeated HBTU was replaced by DIC as a condensation agent. Not more than two repetitions of some steps were made. The first group of 3 compounds, abbreviated **Si6–8** (Table 1), includes 7-mer KLAKLAK and its conjugates with 1,8-naphtalimide and caffeic acid (Figure 4).

We bonded 1,8-naphtalimide to the peptide chain using glycine as a linker in order to evaluate also the manner of bonding to the peptide chain on the biological activity. Thus, 1,8-naphtalimideglycine was synthesized with good yield and purity according to Marinov et al. [44].

The biological activity of the series is tested according to standard protocols for cytotoxicity (BALB/3T3 NRU-test) and antiproliferative activity (MTT dye reduction assay). **Si6** is practically non-toxic and does not show antiproliferative effects on the used cell lines at tested concentrations. The addition of 1,8-naphthalimide to KLAKLAK-NH_2_ (1,8-naphthalimideG-KLAKLAK-NH_2_ or **Si7**) resulted in a slight increase in cytotoxicity and antiproliferative effect in MCF-10A and MCF-7 cell lines. The addition of caffeic acid (**Si8**) resulted in a significant increase in cytotoxicity in BALB/c 3T3 cells (IC_50_ decreased from > 4000 to 710.3 ± 11.91 µM). There was also more than ten-fold increase in antiproliferative activity (IC_50_ from > 2000 to 135.6 ± 7.09, 128.6 ± 8.03 and 514.3 ± 26.82 for MCF-10A, MCF-7 and MDA-MB-231 respectively). The substances **Si6**, **Si7** and **Si8** do not show selectivity with respect to the studied tumor cell lines.

As it was already mentioned above replacement of natural with unnatural amino acids in the primary structure of some peptides often leads to increased activity. Taking into account this fact the second step of this investigation was to replace Ala with β-Ala and Leu with nor-Leu in the KLAKLAK sequence. Thus, a series of six compounds (**Si2,3,11,13,14,15**, Table 1) was synthesized including bioconjugates of newly synthesized peptides again with 1,8-naphtalimide and caffeic acid. Their biological activity was also tested against a panel of normal and tumor cell lines. Substitution of Ala with β-Ala in **Si3** resulted in a slight increase in cytotoxicity in BALB/c 3T3 cells (IC_50_ = 2874 ± 129.5) and antiproliferative effect in MCF-10A cells (IC_50_ = 1469 ± 103.8). In contrast **Si3** had a high antiproliferative effect (IC_50_ = 176.3 ± 4.66) according to MCF-7. The calculated selective index for MCF-7 is significant (SI = IC_50_ MCF-10A/IC_50_MCF-7 = 1469/176.3 = 8.33). Substitution of Leu with nor-Leu in **Si13** did not result in a significant change in biological activity in the cell lines used. The additional group (1,8-naphthalimideG) in **Si2** increases the biological activity but no significant SI is observed. Unlike **Si11**, where the group (Caf-) is added showed an increased antiproliferative effect and a significant selective index relative to MCF-7 cell line (SI = 2.62). **Si15** causes a significantly higher antiproliferative effect than **Si14**. This is probably due to the chemical group Caf- in **Si15**. No selectivity of the effect was observed in **Si14** and **Si15**.

MDA-MB-231 showed increased resistance to all test substances. This is probably due to the damaged mechanisms of apoptosis in these cells.

The obtained results presented in Table 3 show that only shortened analogue KLAKLAK-NH_2_ and its conjugate with 1,8-naphthalimide containing unnatural amino acid nor-Leu (1,8-naphthalimideGKnLAKnLAK-NH_2_) show moderate activity against the *Escherichia coli K12 407* at 20 μM concentration. This result is in agreement with the results of Johnson et al. [50] who also observed antibacterial effect against strains *E. coli* of some tested analogues (KLAKLAK)_2_-NH_2_. The same authors suggested that antibacterial effect is due to cell membrane damage and followed by lysis accelerated by the peptide. Moreover, in accordance with Chan et al. substitution of Leu with nor-Leu could lead to obtaining of good candidates as vectorizing agents with better selectivity and improved antibacterial properties because of better interaction with specific phospholipid membranes [47,48]. Newly obtained analogues were also tested for antimicrobial activity against the model strain Gram-positive microorganisms *Bacillus subtilis 3562* and the model strain fungi *Candida albicans 74*, but any activity was revealed.

## 4. Materials and Methods

### 4.1. Chemical Part

All specifically protected amino acids, Fmoc-Rink Amide MBHA Resin and other reagents and solvents for peptide synthesis were purchased from Iris Biotech (Marktredwitz, Germany). 1,8-naphthalic anhydride is from Sigma-Aldrich (Product of UK). Caffeic acid is from Alfa Aesar (Lancashire, UK).

The purity of newly synthesized compounds were monitored by means of Shimadzu LC MS/MS 8045 system (Shimadzu Corporation, Japan), column Agilent Poroshell 120, 100 mm × 4.6 mm, mobile phase rate 0.30 mL/min, column temperature 40 °C. The following gradient elution was developed: Mobile phase A: H_2_O (10% AcCN; 0.1% HCOOH); Mobile phase B: AcCN (5% H_2_O; 0.1% HCOOH). Gradient of mobile phase start with 80%A/20%B, passes through 5%A/95%B in 15 min and returns to 80%A/20%B in 22 min.

The MS detector is in SCAN regime/ESI+ mode of ionization with 3 L/min of the nebulizing gas flow, 10 L/min of the heating and drying gas flow, 350 °C interface temperature, 200 °C DL temperature and 400 °C heat block temperature.

The optical rotation was measured on automatic standard polarimeter Polamat A, Carl Zeis, Jena (Anton Paar Opto Tec GmbH, Seelze, Germany). Melting points were recorded on standard Kofler hot-stage microscope (Reichert, Austria).

For the synthesis of aimed peptides the conventional solid-phase peptide synthesis (SPPS) by means of Fmoc/OtBu strategy was used. Rink-amide MBHA resin was used as solid-phase carrier. HBTU (3-[Bis(dimethylamino)methyliumyl]-3H-benzotriazol-1-oxide hexafluorophosphate) or DIC (N,N′-Diisopropylcarbodiimide) were used as condensation reagents. Three-functional amino acid Lys was embedded as N^α^-Fmoc-Lys(Boc)-OH. The coupling reactions were performed, using for amino acid/HBTU/HOBt/DIEA/resin a molar ratio 3/3/3/9/1 or amino acid/DIC/resin a molar ratio 3/3/1 and catalytic quantity of 4-N,N-dimethylaminopyridine. The N^α^-Fmoc-group was deprotected on every step by treatment with 20% piperidine solution in N,N′-dimetilformamide (DMF). The coupling and deprotection reactions were checked by both the standard Kaiser and Chloranil test. The releasing of aimed peptides from the resin was done, using a mixture of 95% trifluoroacetic acid (TFA), 2.5% triisopropylsilane (TIS) and 2.5% water. The peptides were obtained as oils in TFA and further precipitated in cold dry diethyl ether. The peptide purity was monitored and their structure was proven on a Shimadzu LC MS/MS 8045 system using the condition described above. The optical rotation was measured in methanol at c = 1. The analytical data for the synthesized peptides are shown in Table 1.

1,8-naphtalimideglycine were synthesized according to Marinov et al. 2020 [44].

### 4.2. Biological Part

#### 4.2.1. In Vitro Cytotoxicity Testing (3T3 NRU Test)

The cytotoxicity testing was performed as described by Borenfreund et al. [51] and the latest modification of the validated BALB/3T3 clone A31 Neutral Red Uptake Assay (3T3 NRU test) [52] for cytotoxicity testing. BALB/3T3, clone A31 mouse embryo cells were grown as monolayer in 75 cm^2^ tissue culture flasks in DMEM high-glucose (4.5 g/L), supplemented with 10% FBS and antibiotics (Sigma-Aldrich, Schnelldorf, Germany). Cultures were maintained at 37.5 °C in a humidified atmosphere under 5% CO_2_. Cells were plated at a density of 1 × 10^4^ cells in 100 μL culture medium in each well of 96-well flat-bottomed microplates (Biologix, Lenexa, KS, USA) and allowed to adhere for 24 h. The test compounds, dissolved in DMSO and diluted in culture medium to concentration range 30 to 4000 μM were then added and the cell cultures were incubated for additional 24 h. A wide concentration range was applied (from 30 to 4000 μM) and the cells were incubated for additional 24 h. After treatment with Neutral Red medium, washing and treatment with the Ethanol/Acetic acid solution (NR Desorb), the absorption was measured on a TECAN microplate reader (TECAN, Grödig, Austria) at wavelength 540 nm.

#### 4.2.2. In Vitro Antiproliferative Activity

The antiproliferative activity testing was performed on cell cultures from several human cell lines using the standard MTT-dye reduction assay, described by Mosmann [53]. The assay is based on the metabolism of the tetrazolium salt MTT to insoluble formazan by mitochondrial reductases. The formazan concentration can be determined spectrophotometrically. The measured absorption is an indicator of cell viability and metabolic activity. Cell lines: mammary gland type A adenocarcinoma ER+, PR+, HER2- (MCF-7), triple-negative breast cancer ER-, PR-, HER2- (MDA-MB-231) and breast, non-tumorigenic epithelial cell line (MCF-10A) were used in experiments. The cell lines were routinely grown as monolayers in 75 cm^2^ tissue culture flasks under standard conditions (described above). Cells were plated at a density of 1 × 10^3^ cells in 100 µL in each well of 96-well flat-bottomed microplates and allowed to adhere for 24 h before treatment with test compounds. A concentration range from 15 to 2000 μM was applied for 72 h. The formazan absorption was registered using a microplate reader at λ = 540 nm. Antiproliferative activities were expressed as IC_50_ values (concentrations required for 50% inhibition of cell growth), calculated using non-linear regression analysis (GraphPad Software, San Diego, CA, USA).

The statistical analysis included application of One-way ANOVA followed by Bonferroni’s post hoc test. *p* < 0.05 was accepted as the lowest level of statistical significance. All results are presented as mean ± SD.

#### 4.2.3. Antibacterial Assay

All newly synthesized derivatives of (KLAKLAK)_2_-NH_2_ at two concentrations 10 μM and 20 μM, were tested against facultative anaerobic gram-negative *Esherichia coli NBIMCC K12 407*. The strains were obtained from the culture collection of Bulgarian National Bank for Industrial Microorganisms and Cell Cultures (Sofia, Bulgaria) and were cultured in Luria-Bertani (LB) medium (Mumbai, India). The microbiological tests were performed using the agar diffusion method. The overnight pure cultures from tested strains were prepared in liquid LB-medium. A single colony was used for inoculating the liquid LB medium in order to maintain initial bacterial concentration of 1 × 10^7^ cfu/mL. 100 µL of bacterial suspensions were seeded on agar plates with solid LB-medium. After 30 min, sterile paper disks 6 mm in diameter were soaked with tested samples in amount of 6 µL and placed on the agar petri dishes surface. The plates were incubated for 24 h at 37 °C. The appeared inhibition zones and their size were measured. The sterile paper disks soaked with water was used as blank. Mean values were calculated by performing the experiments in triplicates.

## 5. Conclusions

The obtained results reveal that the introducing of 1,8-naphthalimideGly- and Caf- increase the cytotoxicity and antiproliferative activity of the peptides but not their selectivity.

A significant selective index is observed only for substances **Si3** and **Si11**. The common chemical structure of these substances is KLβAKLβAK-NH_2_. Therefore, we believe that the component responsible for high biological activity and selectivity is the amino acid β-Ala in the structure of **Si3** and **Si11**. Due to the significantly higher biological activity of **Si1** ((KLAKLAK)_2_-NH_2_) compared to **Si6** (KLAKLAK-NH_2_), we theoretically predict high antitumor activity and selectivity of the synthetic peptide (KLβAKLβAK)_2_-NH_2_. Only two of tested compounds **Si6** (KLAKLAK-NH_2_) and **Si14** (1,8-naphthalimideGKnLAKnLAK-NH_2_) show moderate activity against the model strain Gram-negative microorganisms *Escherichia coli K12* at low concentration of 20 μM.

## Figures and Tables

**Table 1:** Structure and analytical data for newly synthesized compounds.

Abb	Peptide Structure	Molecular Formula	Mw_exact_	[M + H]^+^ Observed	[M + Na]^+^ Observed	t_R_ (min)	M.p. [°C]	$\alpha_{546}^{20}\;[{}^{\circ}]\;*$	Chromatographic Purity (%)
**Si1**	(KLAKLAK)_2_-NH_2_	C_71_H_135_N_21_O_15_	1522.05	1523.30	-	3.53	118	−85	100.00
**Si6**	KLAKLAK-NH_2_	C_35_H_67_N_11_O_8_	769.52	770.65	-	2.49	136	−33	100.00
**Si7**	1,8-naphthalimideG-KLAKLAK-NH_2_	C_49_H_74_N_12_O_11_	1006.56	1007.70	-	3.44	89	−98	98.07
**Si8**	Caf-KLAKLAK-NH_2_	C_45_H_77_N_11_O_10_	931.59	932.70	956.70	3.72	154	−35	82.37
**Si3**	KLβAKLβAK-NH_2_	C_35_H_67_N_11_O_8_	769.52	770.70	-	2.48	123	−98	99.14
**Si2**	1,8-naphthalimideG-KLβAKLβAK-NH_2_	C_49_H_74_N_12_O_11_	1006.56	1007.80	1029.75	1.48	98	−156	100.00
**Si11**	Caf-KLβAKLβAK-NH_2_	C_45_H_77_N_11_O_10_	931.59	932.65	-	3.65	125	−23	82.17
**Si13**	KnLAKnLAK-NH_2_	C_35_H_67_N_11_O_8_	769.52	770.80	-	1.25	93	−34	100.00
**Si14**	1,8-naphthalimideG-KnLAKnLAK-NH_2_	C_49_H_74_N_12_O_11_	1006.56	1007.75	1029.75	3.42	a **	−12	87.48
**Si15**	Caf-KnLAKnLAK-NH_2_	C_45_H_77_N_11_O_10_	931.59	932.75	-	1.33	92	−64	100.00

* methanol (c = 1); ** a-amorphous.

**Table 2:** Cytotoxic and antiproliferative potency of the studied substances expressed by IC_50_ values of the mean ± SD.

Abb	Peptide Structure	IC_50_ of Mean ± SD (μM)			
		Cytotoxicity	Antiproliferative Activity		
		BALB/3T3	MCF-10A	MCF-7	MDA-MB-231
**Si1**	(KLAKLAK)_2_-NH_2_	365.3 ± 4.08	154 ± 6.53	124.1 ± 8.12	746.5 ± 7.6
**Si6**	KLAKLAK-NH_2_	>4000	>2000	>2000	>2000
**Si7**	1,8-naphthalimideG-KLAKLAK-NH_2_	3422 ± 51.26	1144 ± 64.53	1195 ± 131.5	>2000
**Si8**	Caf-KLAKLAK-NH_2_	710.3 ± 11.91	135.6 ± 7.09	128.6 ± 8.03	514.3 ± 26.82
**Si3**	KLβAKLβAK-NH_2_	2874 ± 129.5	1469 ± 103.8	176.3 ± 4.66	>2000
**Si2**	1,8-naphthalimideG-KLβAKLβAK-NH_2_	1429 ± 48.38	666 ± 20.89	662.9 ± 20.02	840 ± 21.18
**Si11**	Caf-KLβAKLβAK-NH_2_	742.5 ± 18.49	597.2 ± 53.05	228.8 ± 7.18	1087 ± 70.71
**Si13**	KnLAKnLAK-NH_2_	>4000	>2000	1704 ± 112	>2000
**Si14**	1,8-naphthalimideG-KnLAKnLAK-NH_2_	2893 ± 61.38	630.8 ± 51.16	593.3 ± 60.3	1049 ± 49.77
**Si15**	Caf-KnLAKnLAK-NH_2_	1514 ± 12.16	146.8 ± 7.96	140.3 ± 7.12	346.3 ± 7.91
Cisplatin *		>100	46.89 ± 19.85	19.85 ± 3.74	1.833 ± 0.13

* positive control.

**Table 3:** Average inhibition zones size (mm) formed around compounds at concentration 20 μM against *Escherichia coli K12 407*.

Abb	Peptide Structure	Average Value [mm] *
**Si1**	(KLAKLAK)_2_-NH_2_ (control compound)	5 ± 0.15
**Si6**	KLAKLAK-NH_2_	9.7 ± 0.25
**Si7**	1,8-naphthalimideG-KLAKLAK-NH_2_	0
**Si8**	Caf-KLAKLAK-NH_2_	5 ± 0.15
**Si3**	KLβAKLβAK-NH_2_	0
**Si2**	1,8-naphthalimideG-KLβAKLβAK-NH_2_	0
**Si11**	Caf-KLβAKLβAK-NH_2_	0
**Si13**	KnLAKnLAK-NH_2_	0
**Si14**	1,8-naphthalimideG-KnLAKnLAK-NH_2_	11.7 ± 0.3
**Si15**	Caf-KnLAKnLAK-NH_2_	0

* Data are means of three replicates ± SD.

## Data Availability

Data is contained within the article or supplementary material.

## Supplementary Materials

The following are available online.

## Sample Availability

Limited amounts of the compounds are available from the authors of UCTM.
