# Supplementary material for: Synthesis, Antitumor and Antibacterial Studies of New Shortened Analogues of (KLAKLAK)2-NH2 and Their Conjugates Containing Unnatural Amino Acids
Source: Molecules. 2021 Feb 8;26(4):898. doi: 10.3390/molecules26040898 (PMC7915940; doi:10.3390/molecules26040898)
Supplement: Supplementary file 1 [file molecules-26-00898-s001.pdf]

Si1

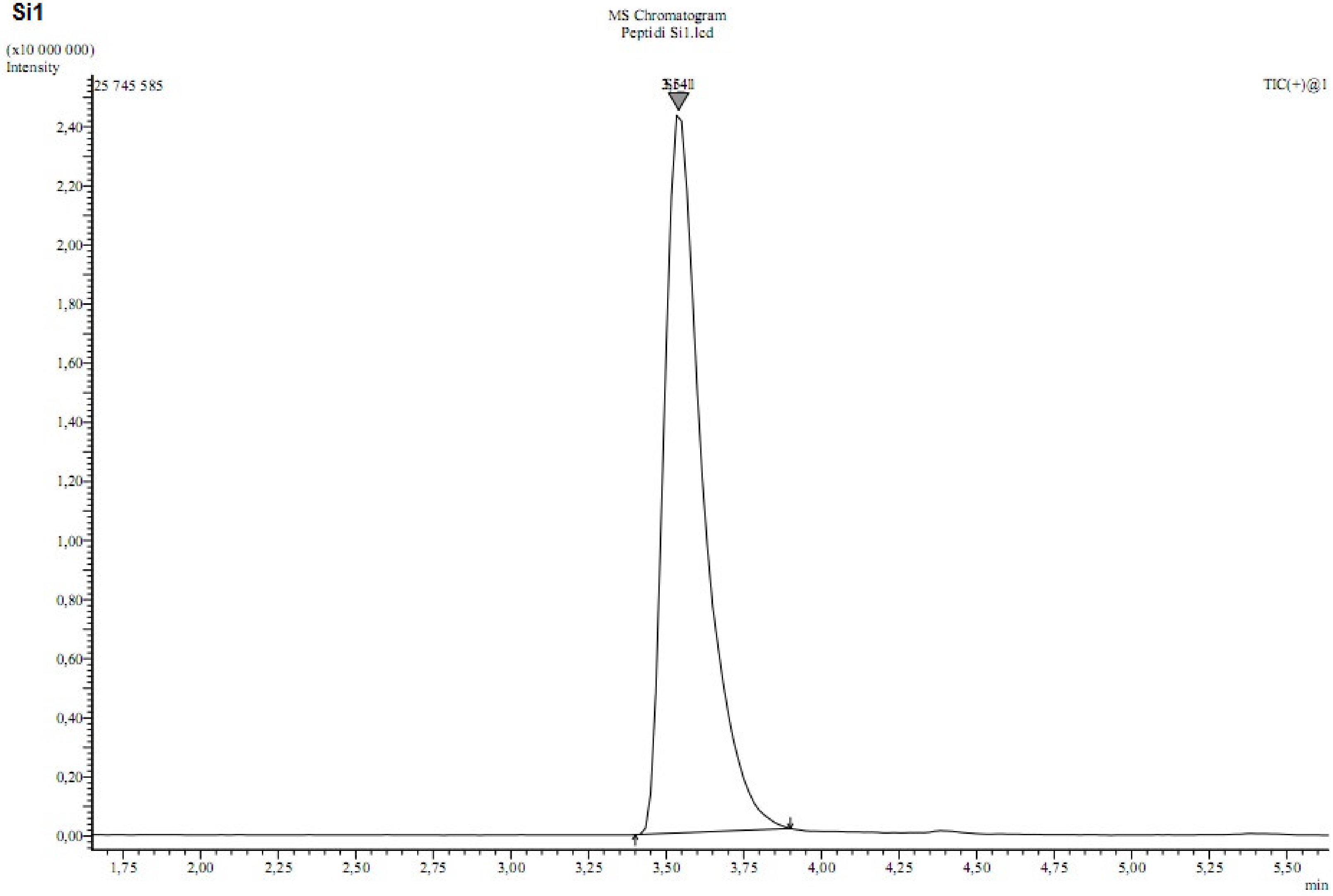
MS Chromatogram
Peptidi Si1.lcd
(x10 000 000)
Intensity
25 745 585
TIC(+)@1
2,40
2,20
2,00
1,80
1,60
1,40
1,20
1,00
0,80
0,60
0,40
0,20
0,00
1,75
2,00
2,25
2,50
2,75
3,00
3,25
3,50
3,75
4,00
4,25
4,50
4,75
5,00
5,25
5,50
min

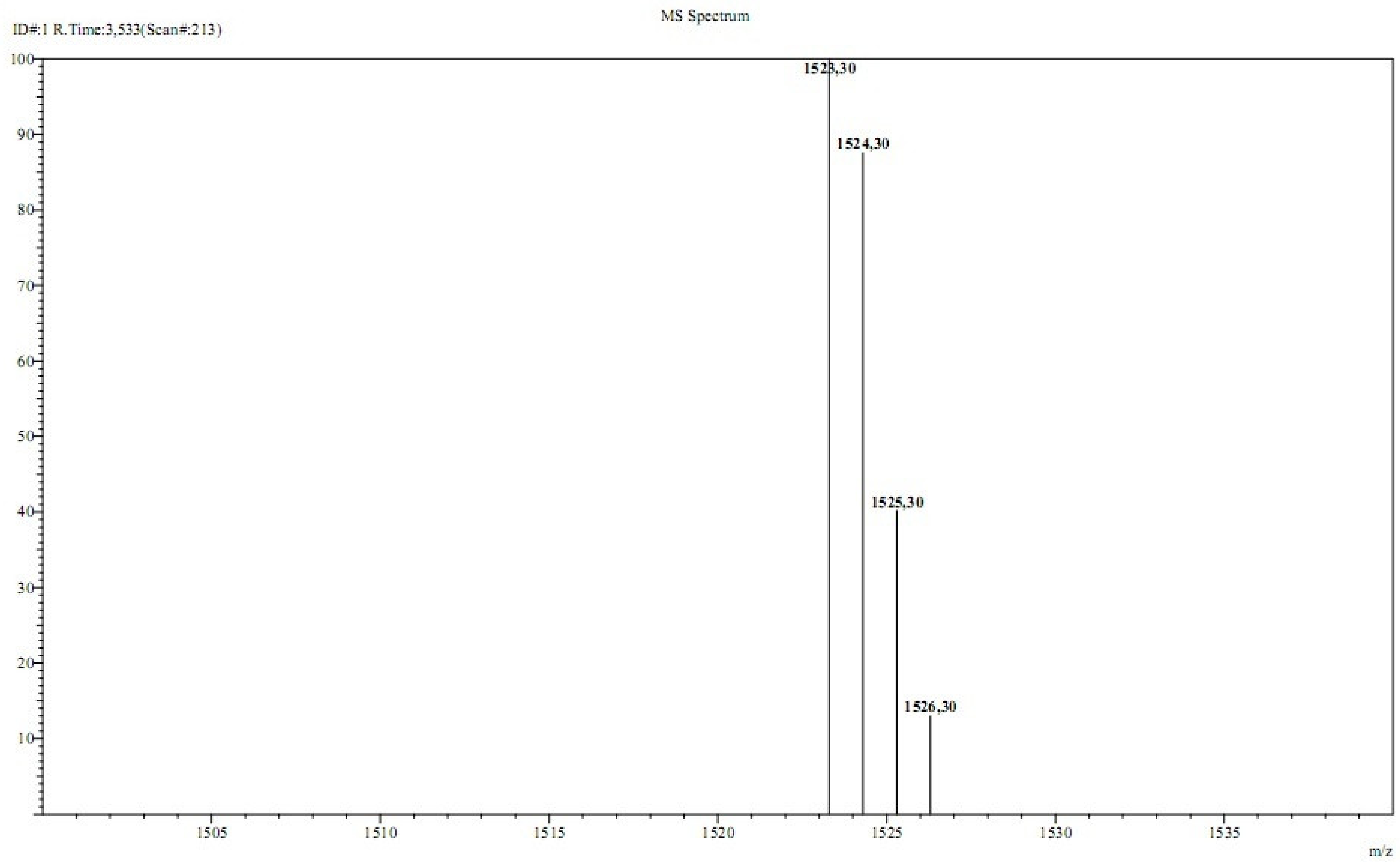
MS Spectrum
ID#:1 R.Time:3,533(Scan#:213)
1523,30
1524,30
1525,30
1526,30
100
90
80
70
60
50
40
30
20
10
1505
1510
1515
1520
1525
1530
1535
m/z

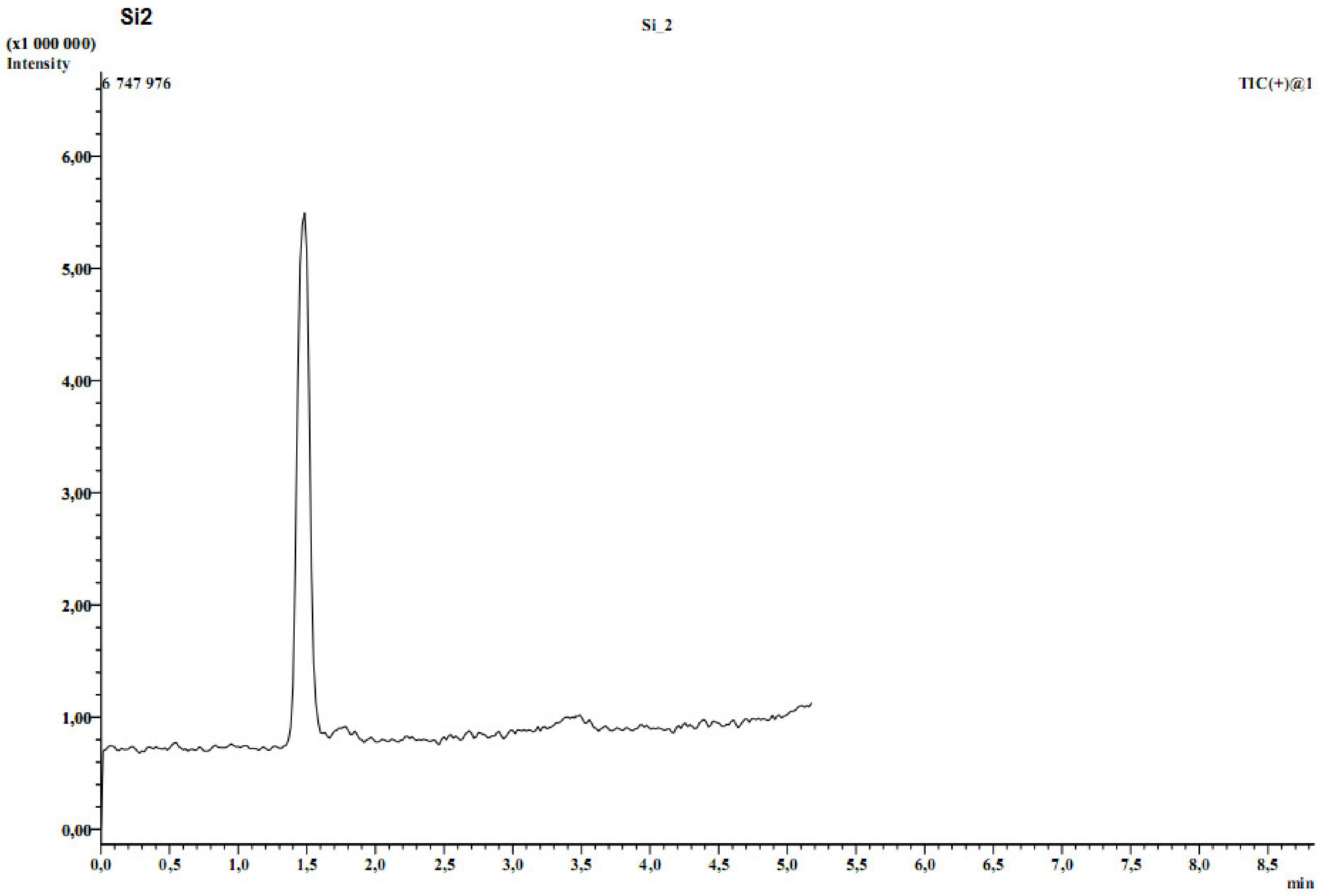
Si2
Si_2
(x1 000 000)
Intensity
6 747 976
TIC(+)@1
6,00
5,00
4,00
3,00
2,00
1,00
0,00
0,0 0,5 1,0 1,5 2,0 2,5 3,0 3,5 4,0 4,5 5,0 5,5 6,0 6,5 7,0 7,5 8,0 8,5
min

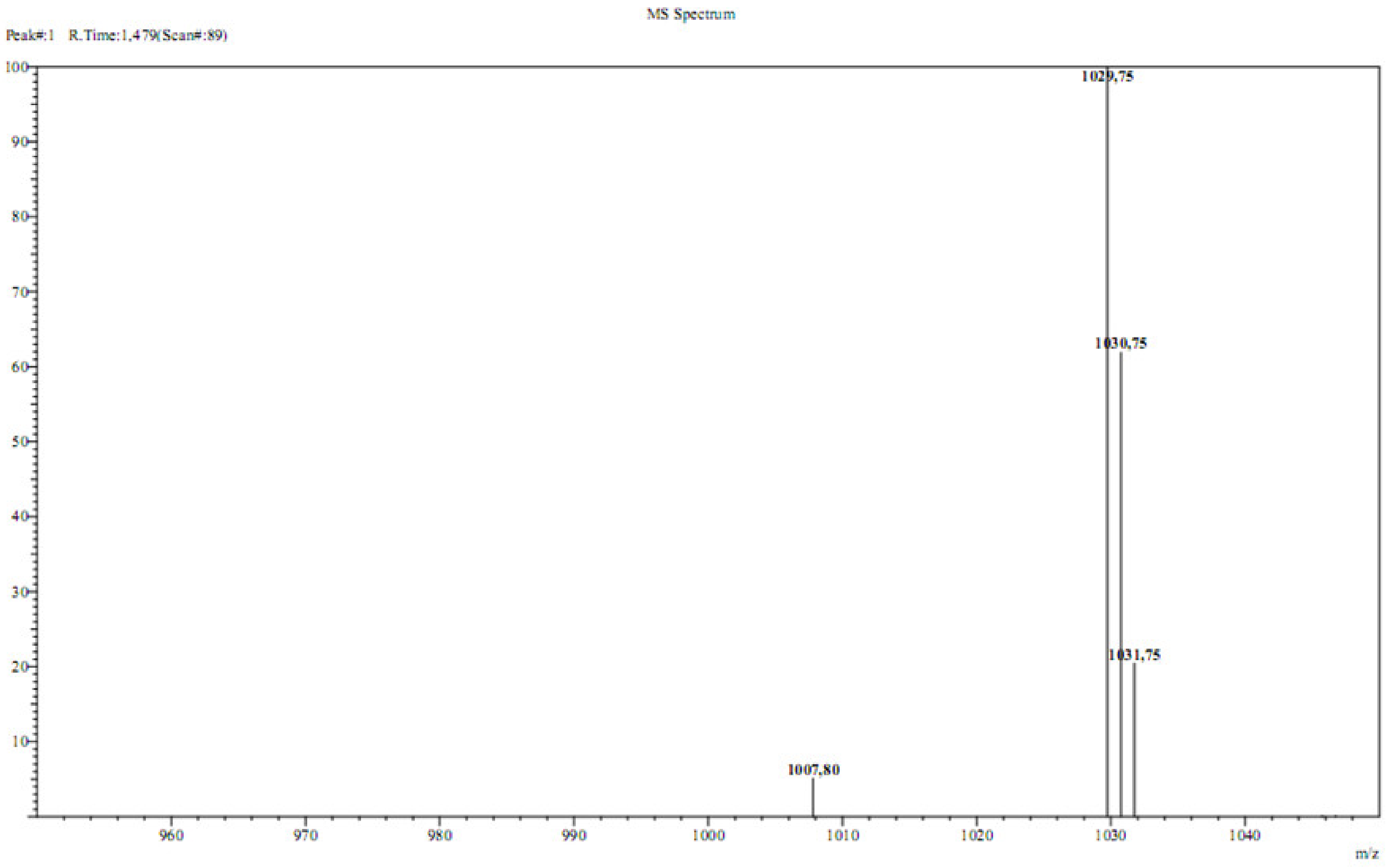
MS Spectrum
Peak#:1 R.Time:1,479(Scan#:89)
1029,75
1030,75
1031,75
1007,80
m/z

Si3

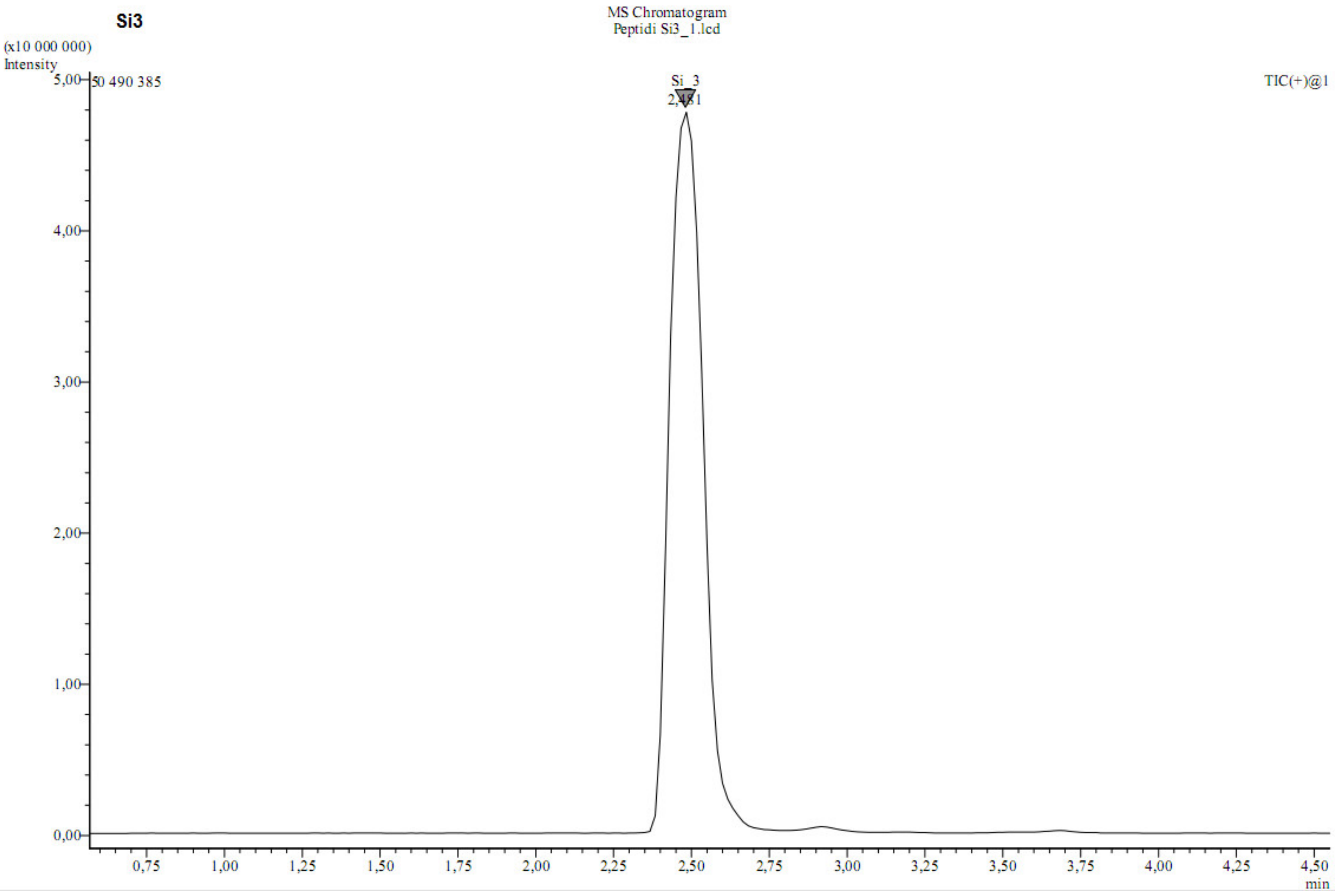

MS Chromatogram
Peptidi Si3_1.lcd
(x10 000 000)
Intensity
50 490 385
Si_3
TIC(+)@1
5,00
4,00
3,00
2,00
1,00
0,00
0,75
1,00
1,25
1,50
1,75
2,00
2,25
2,50
2,75
3,00
3,25
3,50
3,75
4,00
4,25
4,50
min

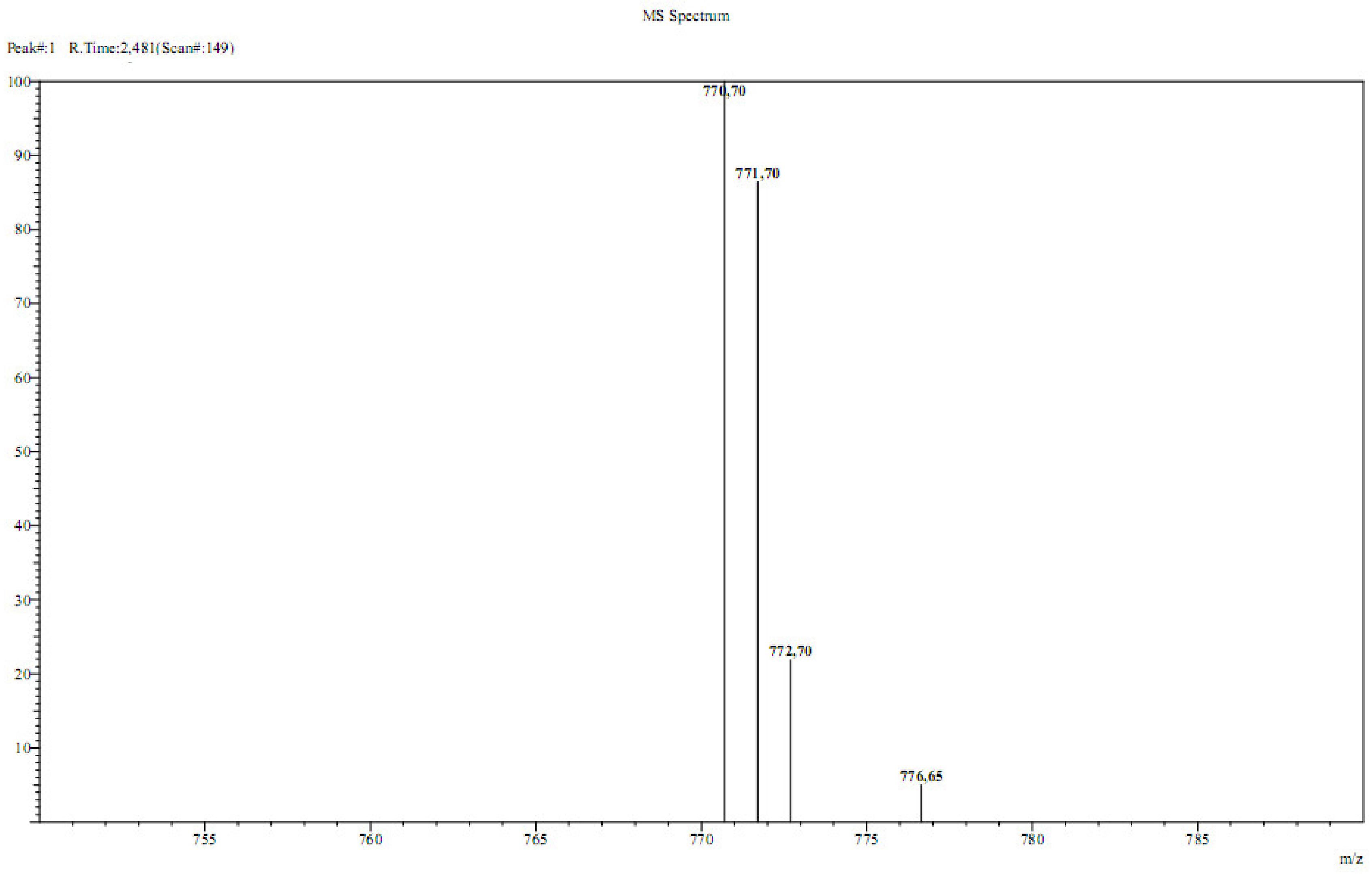
MS Spectrum
Peak#:1 R.Time:2,481(Scan#:149)
100
90
80
70
60
50
40
30
20
10
770,70
771,70
772,70
776,65
755
760
765
770
775
780
785
m/z

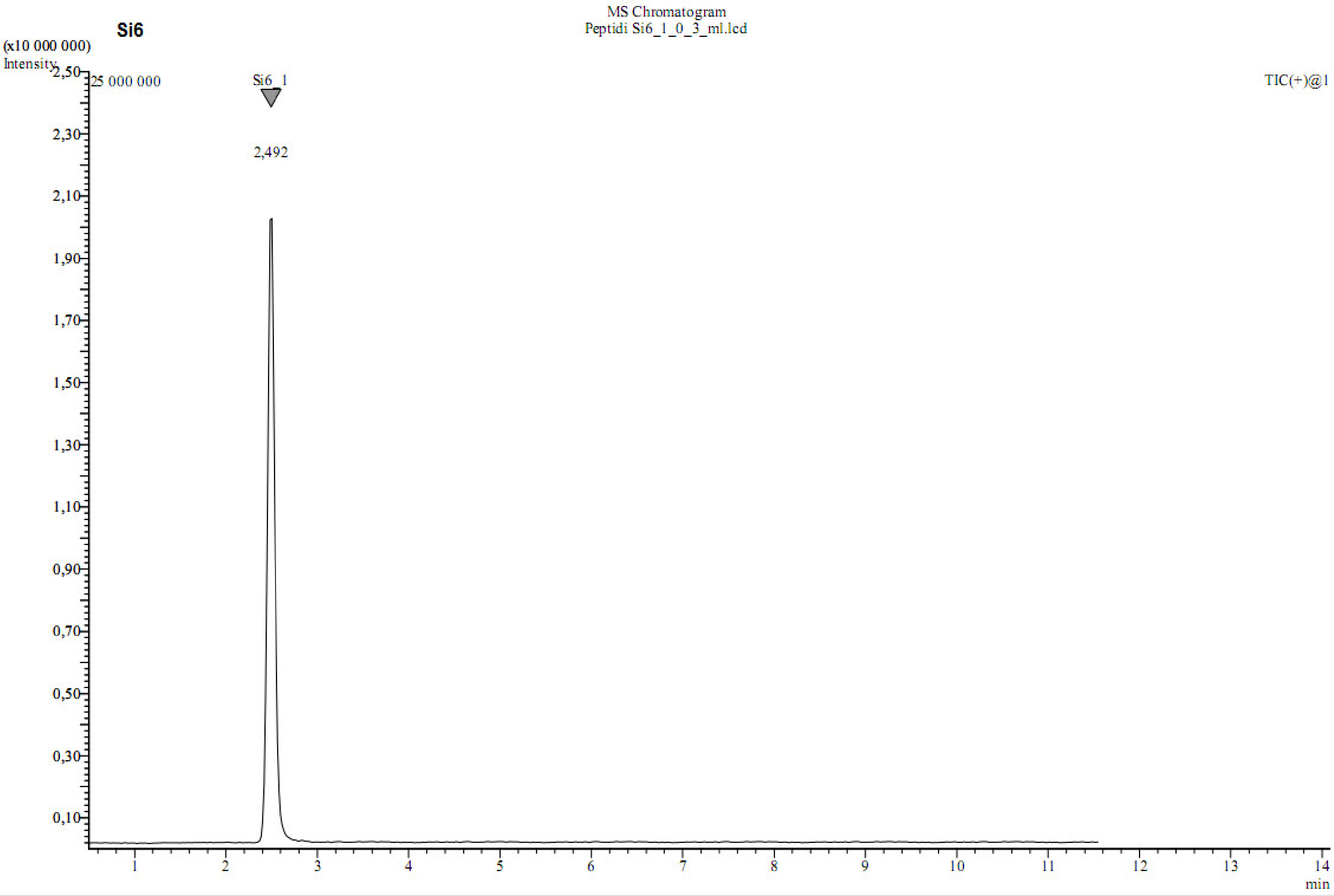
MS Chromatogram
Peptidi Si6_1_0_3_ml.lcd
Si6
(x10 000 000)
Intensity
2,50
25 000 000
Si6_1
TIC(+)@1
2,492
2,30
2,10
1,90
1,70
1,50
1,30
1,10
0,90
0,70
0,50
0,30
0,10
1
2
3
4
5
6
7
8
9
10
11
12
13
14
min

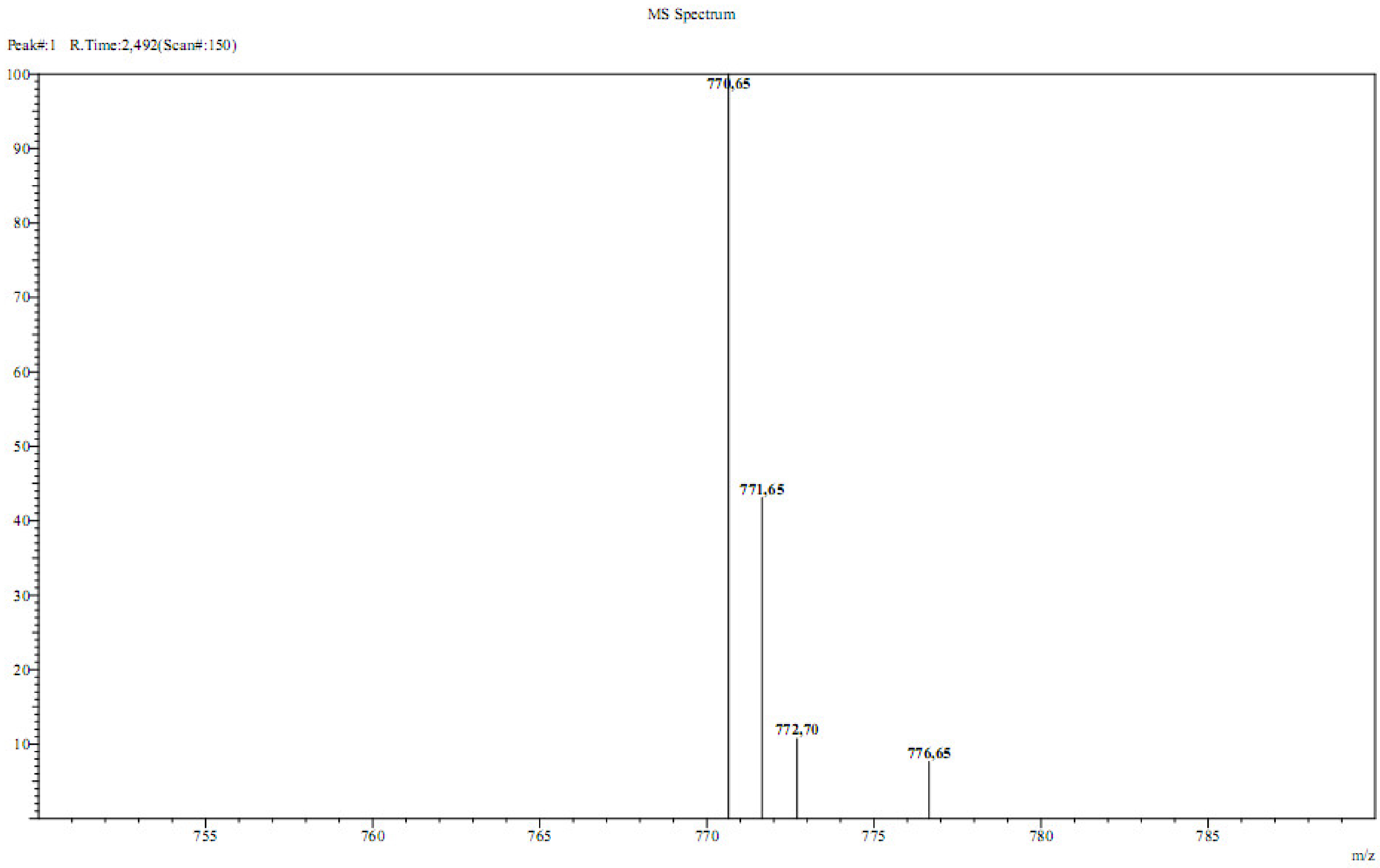
MS Spectrum
Peak#:1 R.Time:2,492(Scan#:150)
100
90
80
70
60
50
40
30
20
10
770,65
771,65
772,70
776,65
755
760
765
770
775
780
785
m/z

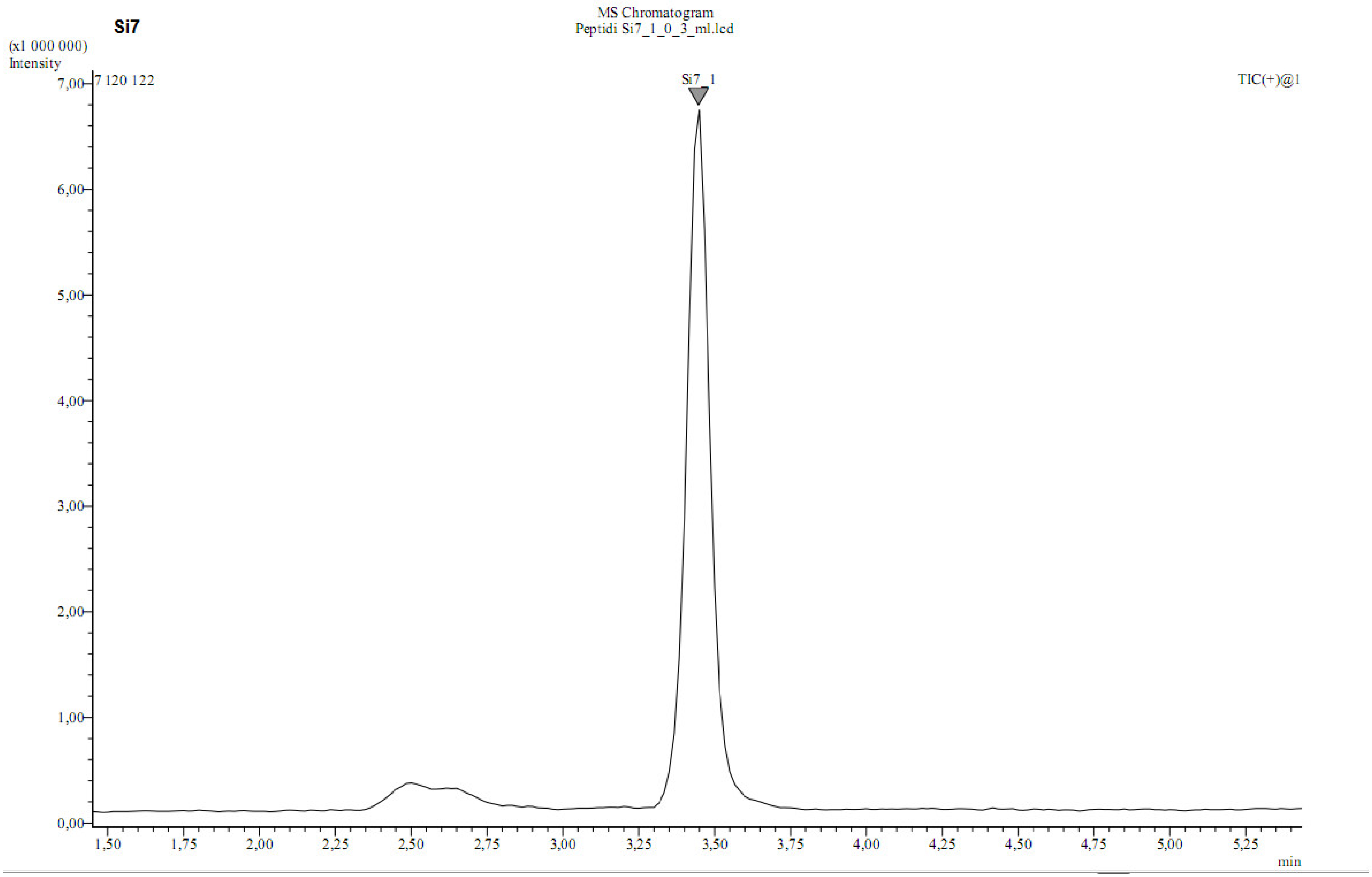
Si7
MS Chromatogram
Peptidi Si7_1_0_3_ml.lcd
(x1 000 000)
Intensity
7 120 122
Si7_1
TIC(+)@1
7,00
6,00
5,00
4,00
3,00
2,00
1,00
0,00
1,50
1,75
2,00
2,25
2,50
2,75
3,00
3,25
3,50
3,75
4,00
4,25
4,50
4,75
5,00
5,25
min

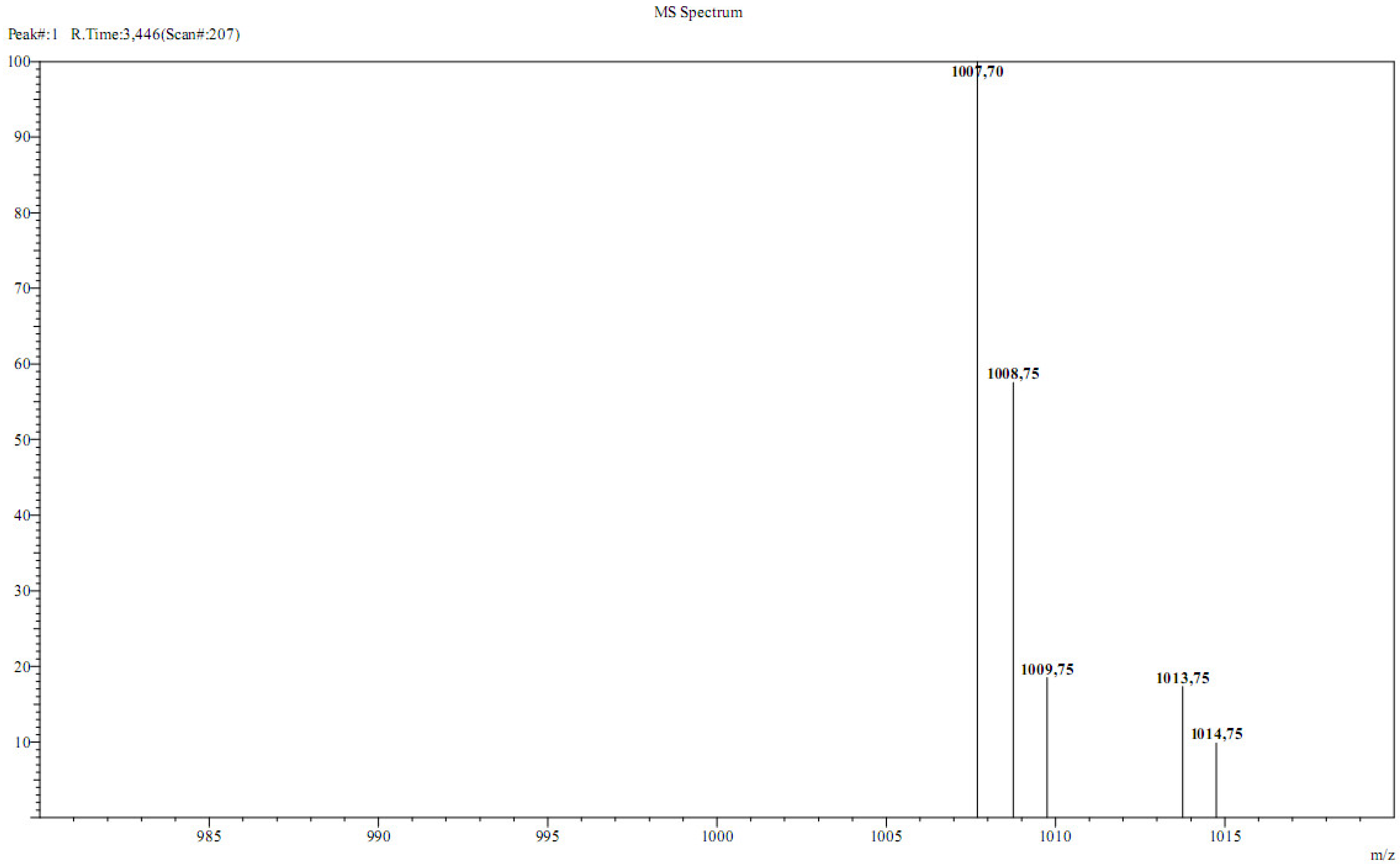
MS Spectrum
Peak#:1 R.Time:3,446(Scan#:207)
1007,70
1008,75
1009,75
1013,75
1014,75
m/z

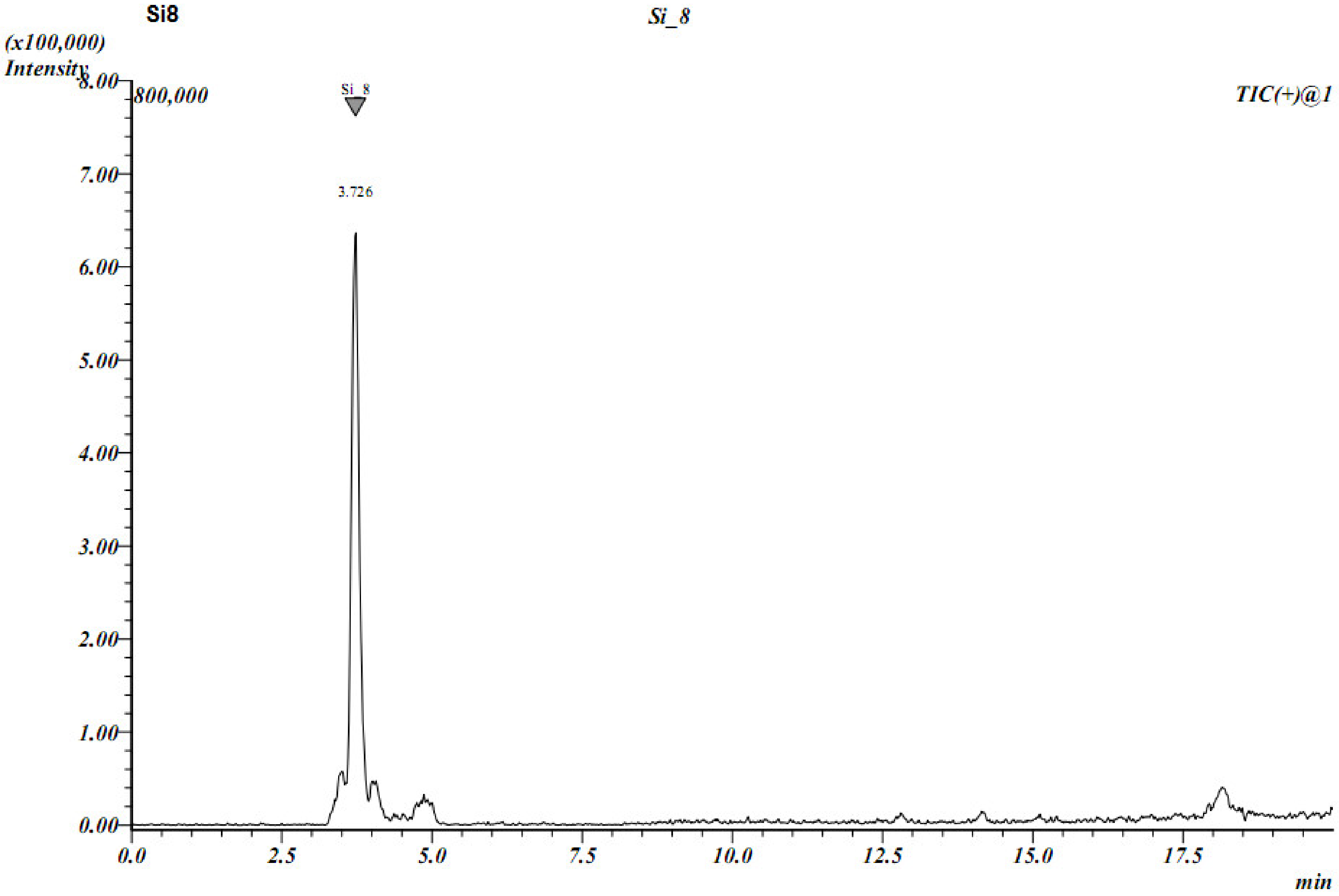

Si8
Si_8
(x100,000)
Intensity
800,000
TIC(+)@1
Si_8
3.726
8.00
7.00
6.00
5.00
4.00
3.00
2.00
1.00
0.00
0.0
2.5
5.0
7.5
10.0
12.5
15.0
17.5
min

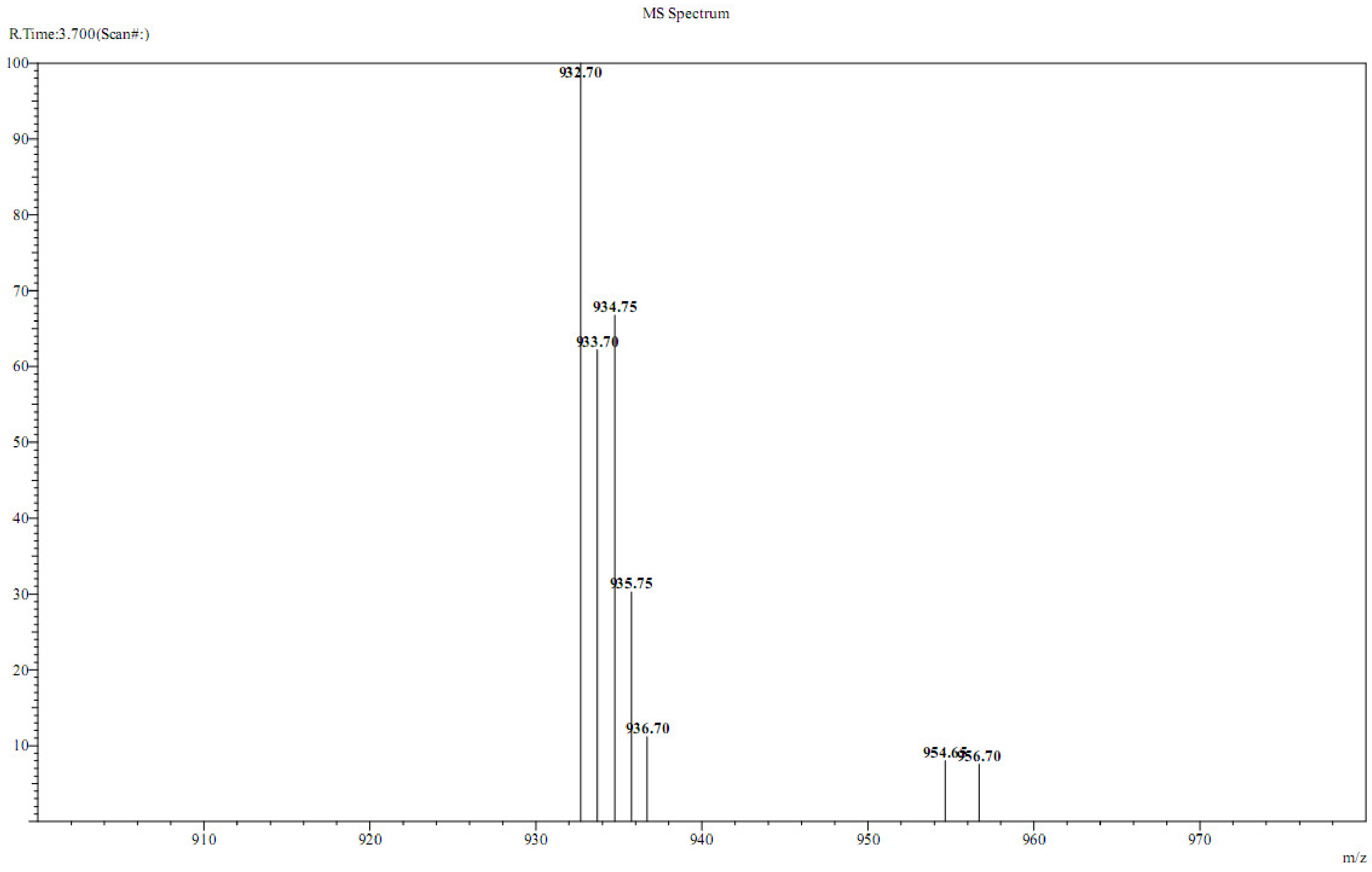

MS Spectrum
R.Time:3.700(Scan#:)
932.70
934.75
933.70
935.75
936.70
954.65
956.70
100
90
80
70
60
50
40
30
20
10
910
920
930
940
950
960
970
m/z

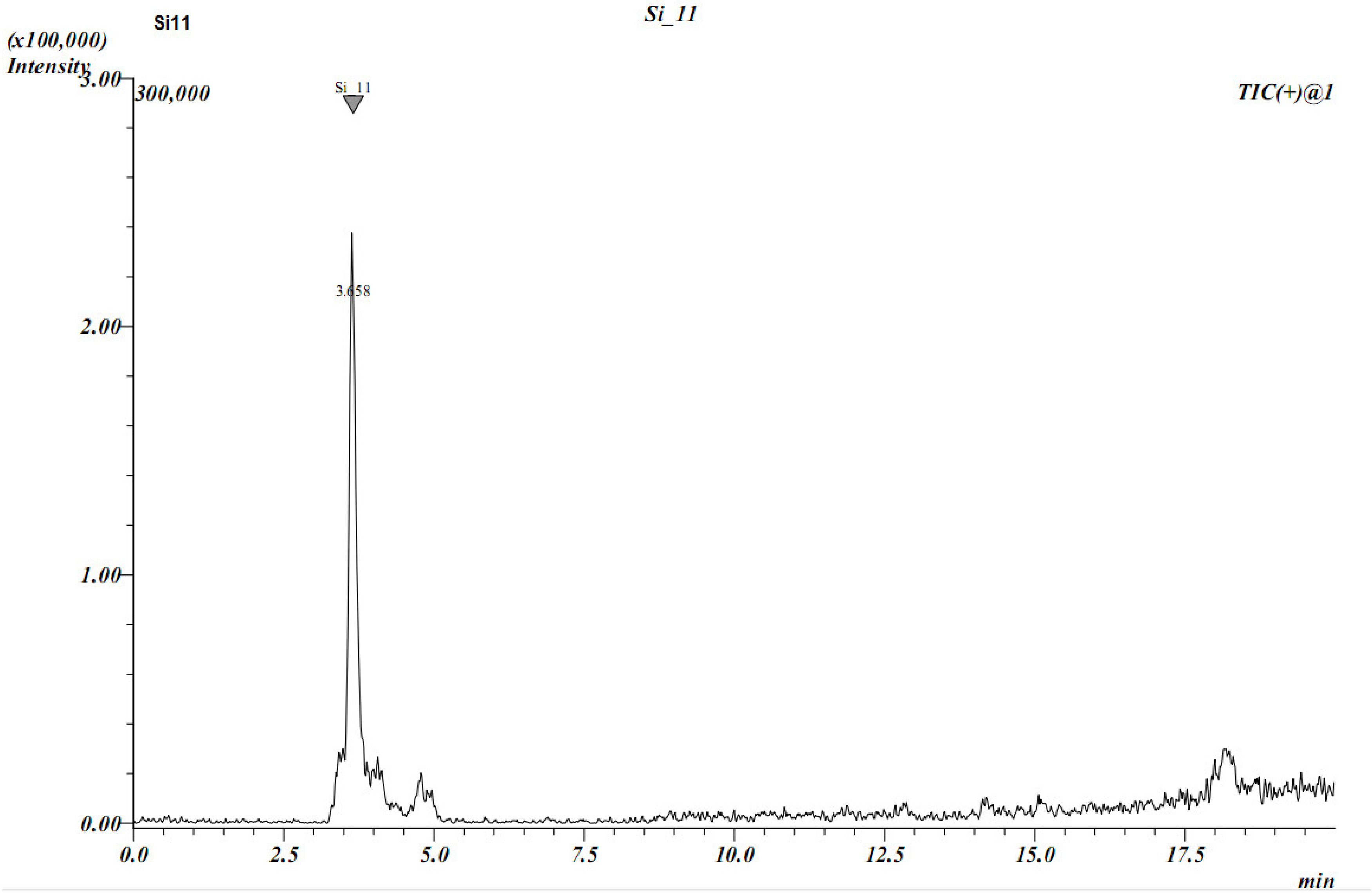

Si11
Si_11
(x100,000)
Intensity
3.00
300,000
Si_11
TIC(+)@1
3.658
2.00
1.00
0.00
0.0
2.5
5.0
7.5
10.0
12.5
15.0
17.5
min

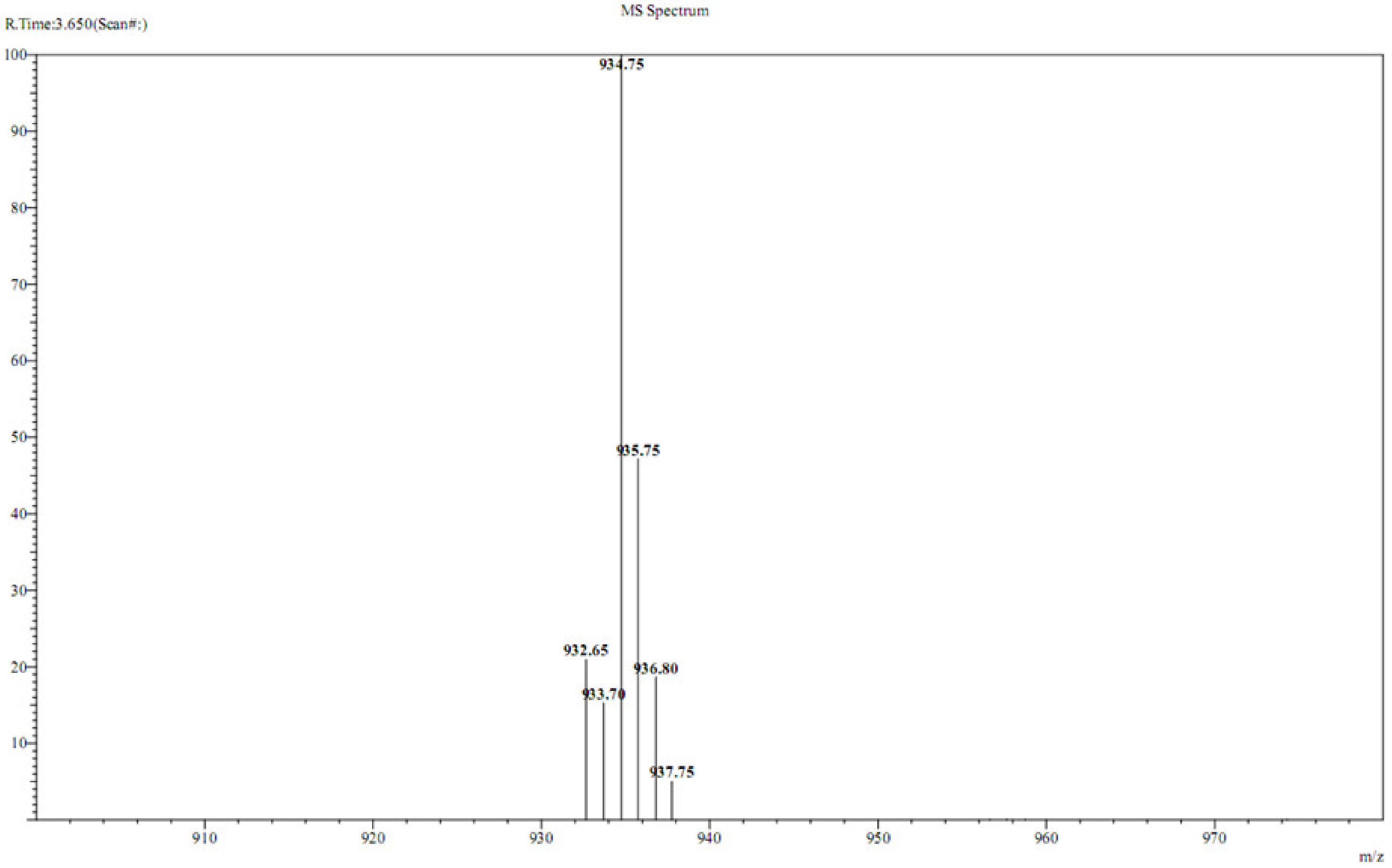
MS Spectrum
R.Time:3.650(Scan#:)
934.75
935.75
932.65
933.70
936.80
937.75
m/z

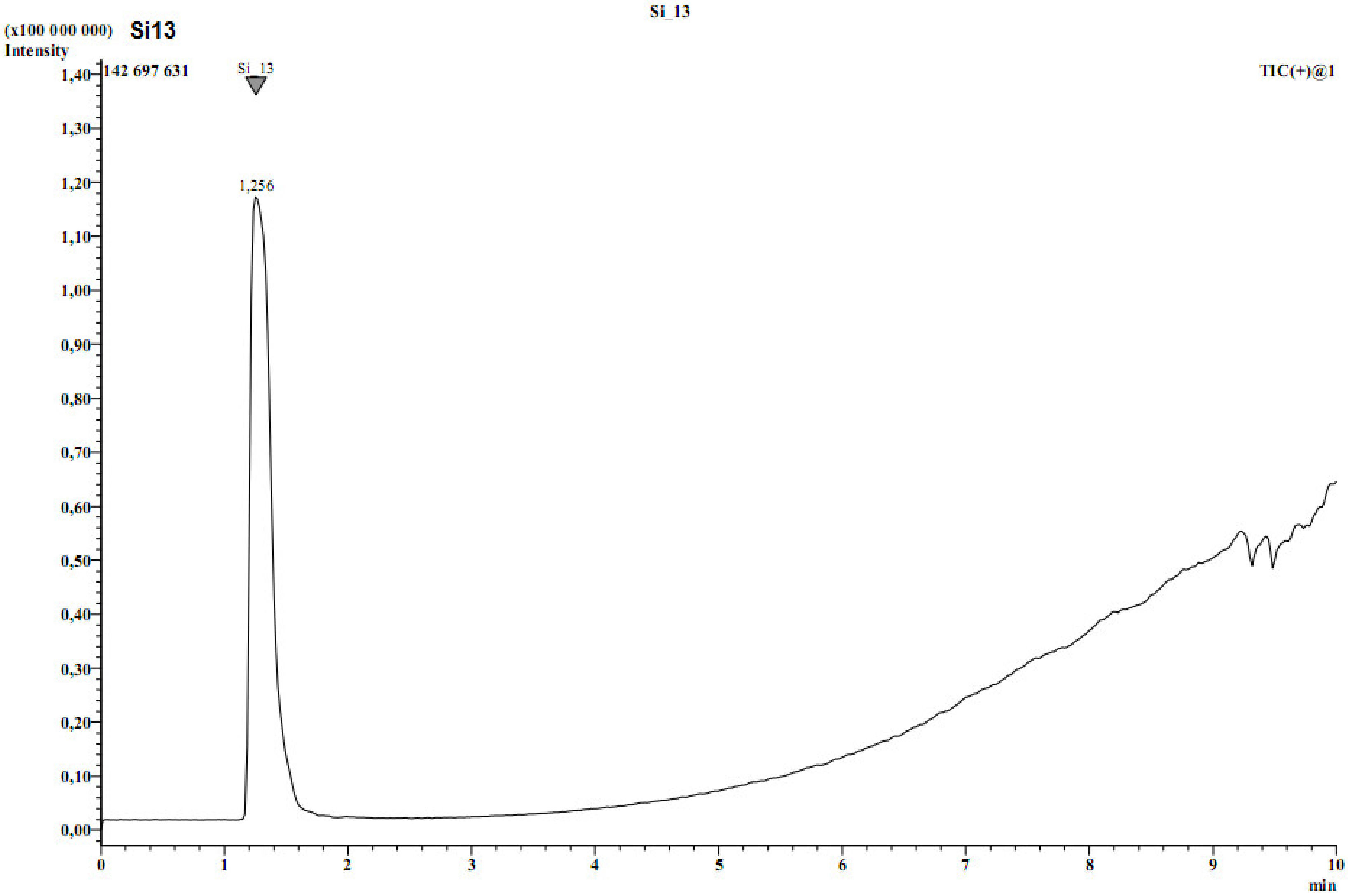
Si_13
(x100 000 000)
Si13
Intensity
142 697 631
Si_13
TIC(+)@1
1,256
1,40
1,30
1,20
1,10
1,00
0,90
0,80
0,70
0,60
0,50
0,40
0,30
0,20
0,10
0,00
0
1
2
3
4
5
6
7
8
9
10
min

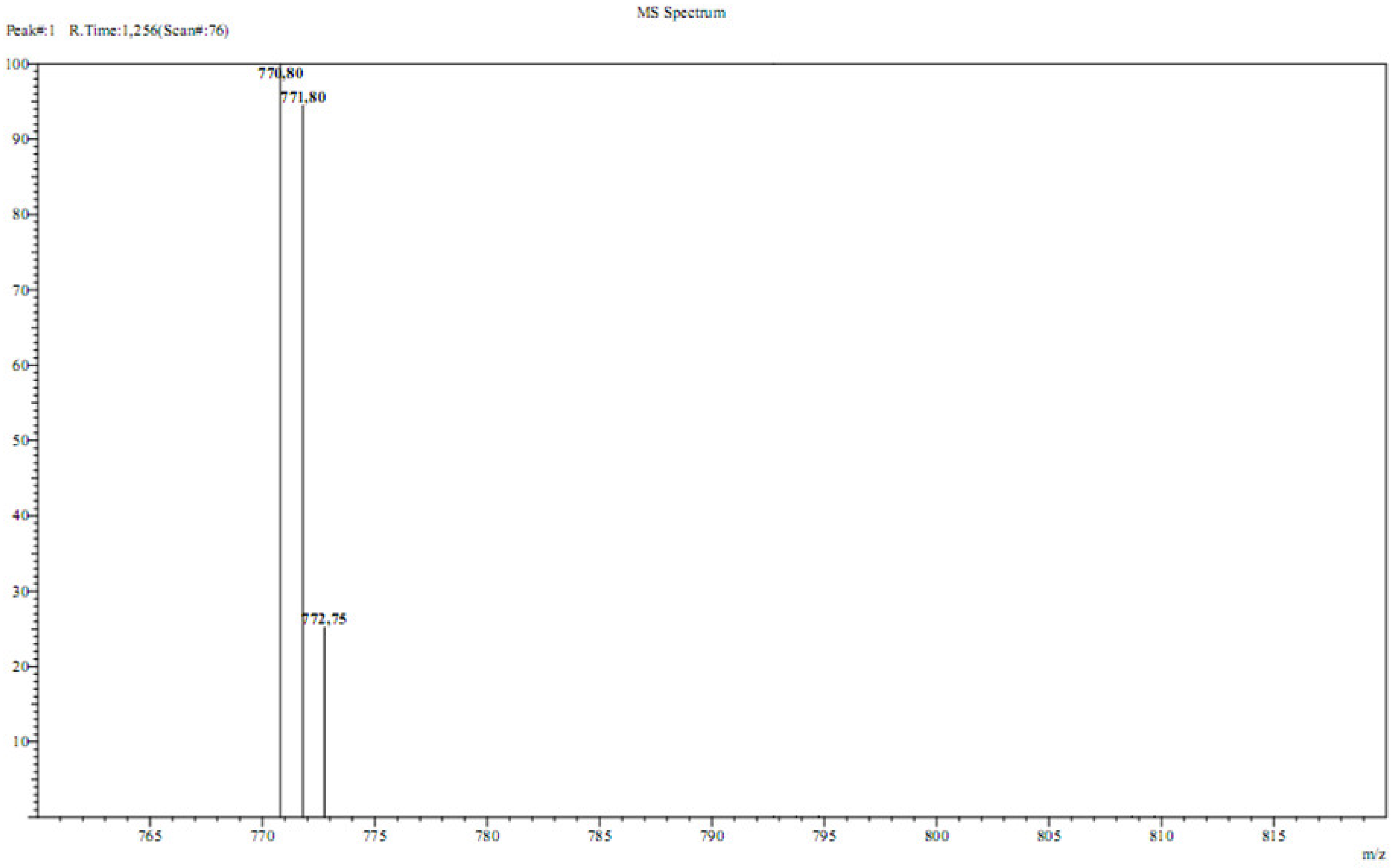
MS Spectrum
Peak#:1 R.Time:1,256(Scan#:76)
770,80
771,80
772,75
100
90
80
70
60
50
40
30
20
10
765
770
775
780
785
790
795
800
805
810
815
m/z

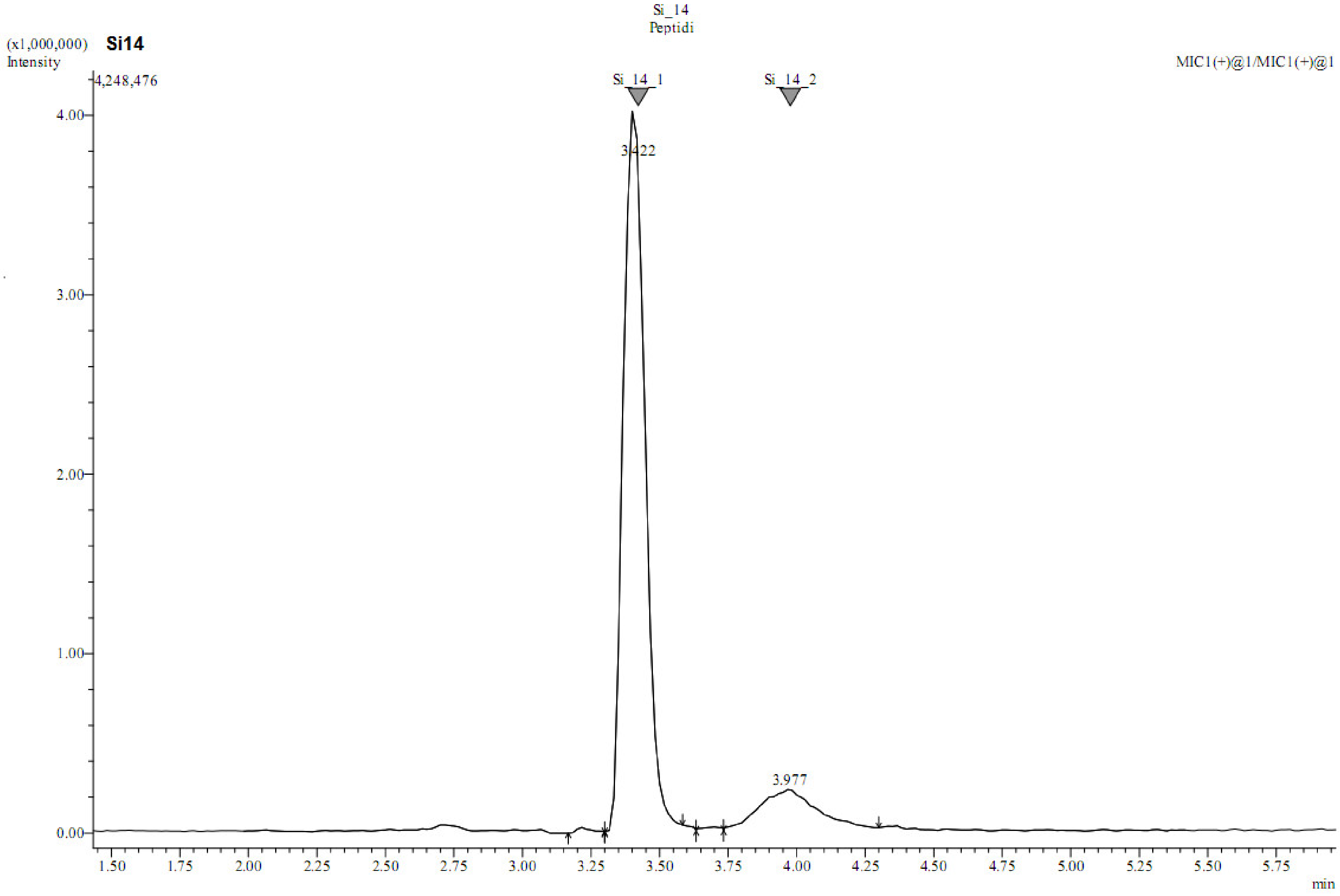

Si_14
Peptidi
(x1,000,000)
Intensity
Si14
MIC1(+)@1/MIC1(+)@1
4,248,476
Si_14_1
Si_14_2
3.422
3.977
4.00
3.00
2.00
1.00
0.00
1.50
1.75
2.00
2.25
2.50
2.75
3.00
3.25
3.50
3.75
4.00
4.25
4.50
4.75
5.00
5.25
5.50
5.75
min

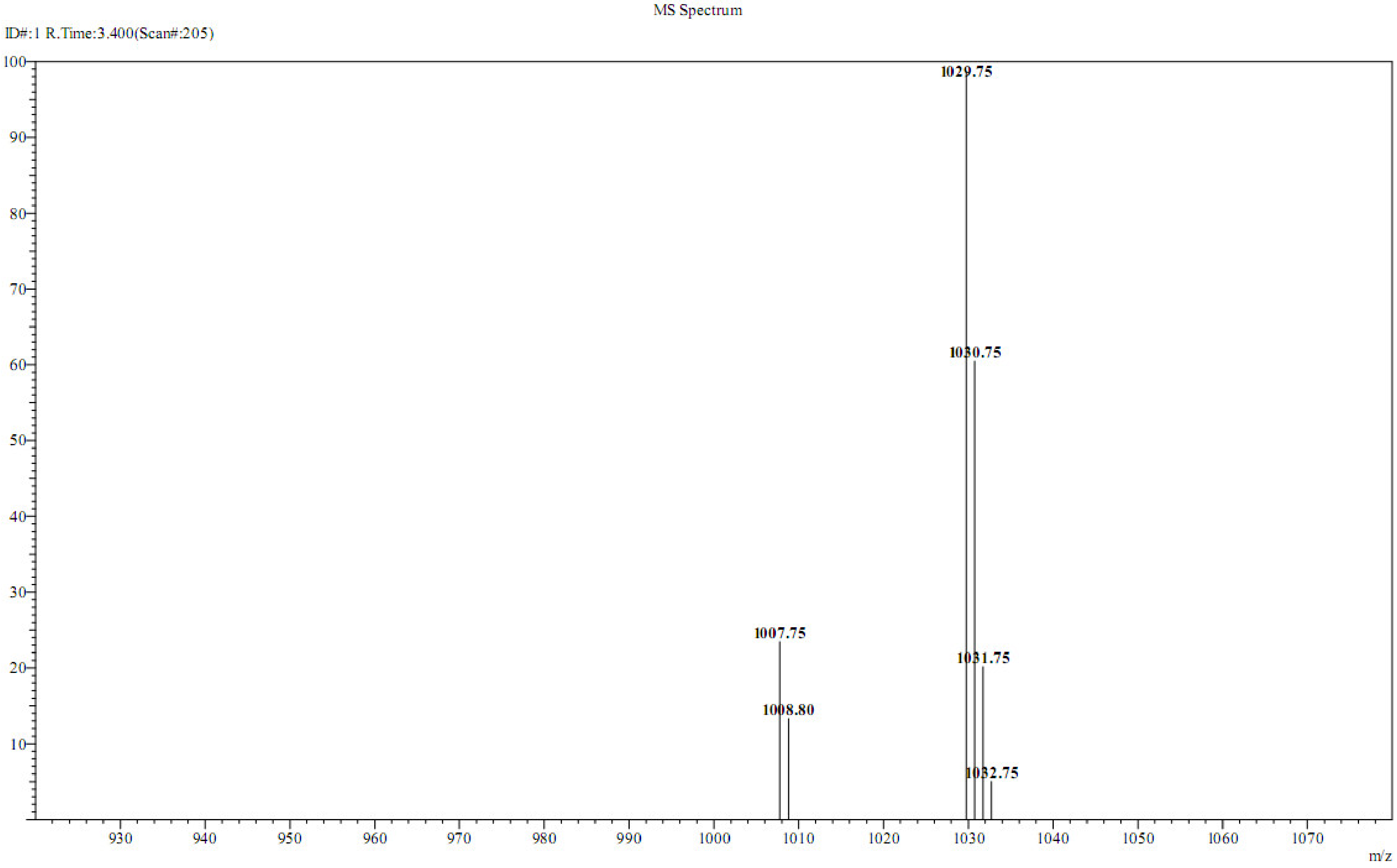
MS Spectrum
ID#:1 R.Time:3.400(Scan#:205)
1029.75
1030.75
1007.75
1031.75
1008.80
1032.75
m/z

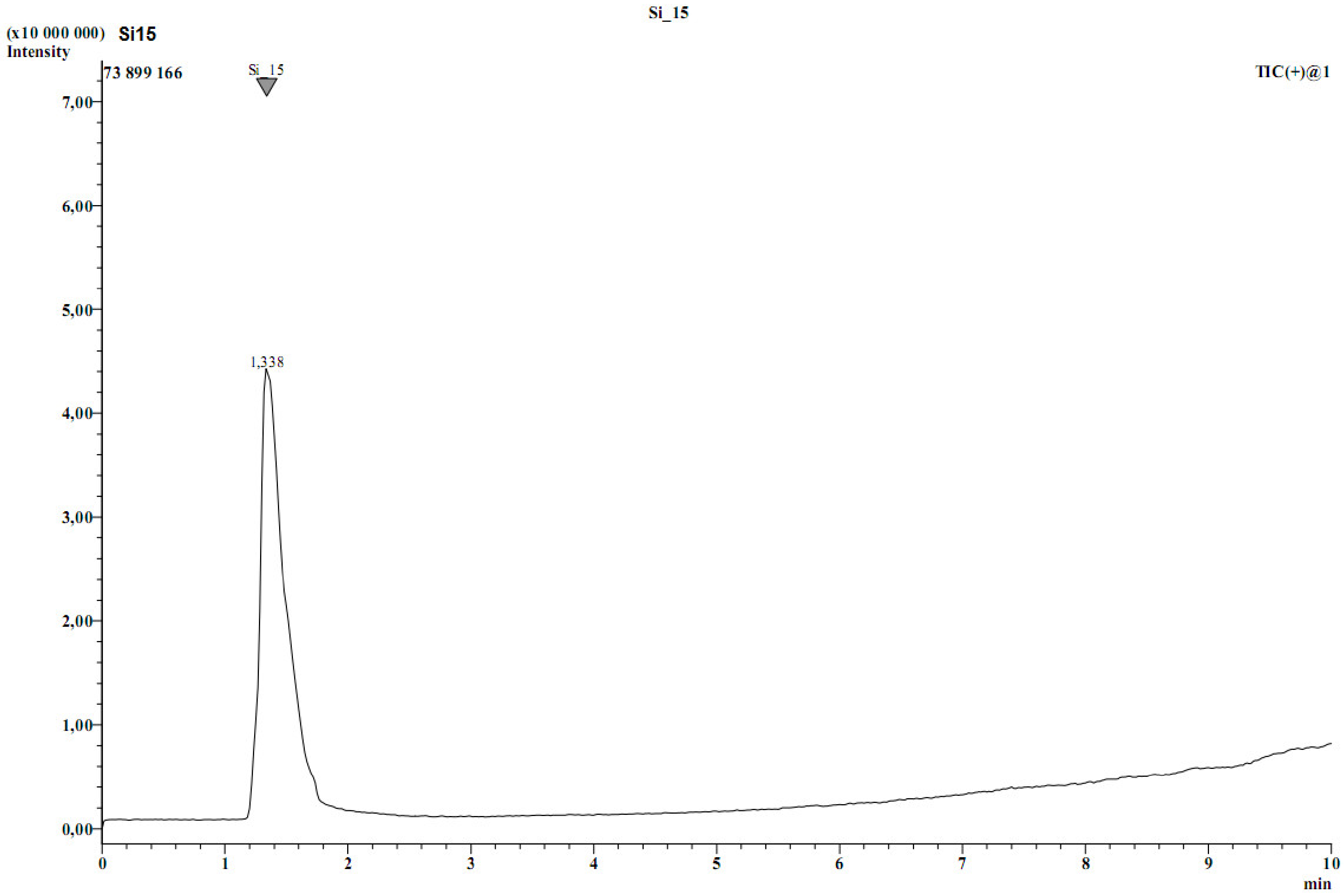
Si_15
(x10 000 000) Si15
Intensity
73 899 166
Si_15
TIC(+)@1
7,00
6,00
5,00
4,00
3,00
2,00
1,00
0,00
1,338
0
1
2
3
4
5
6
7
8
9
10
min

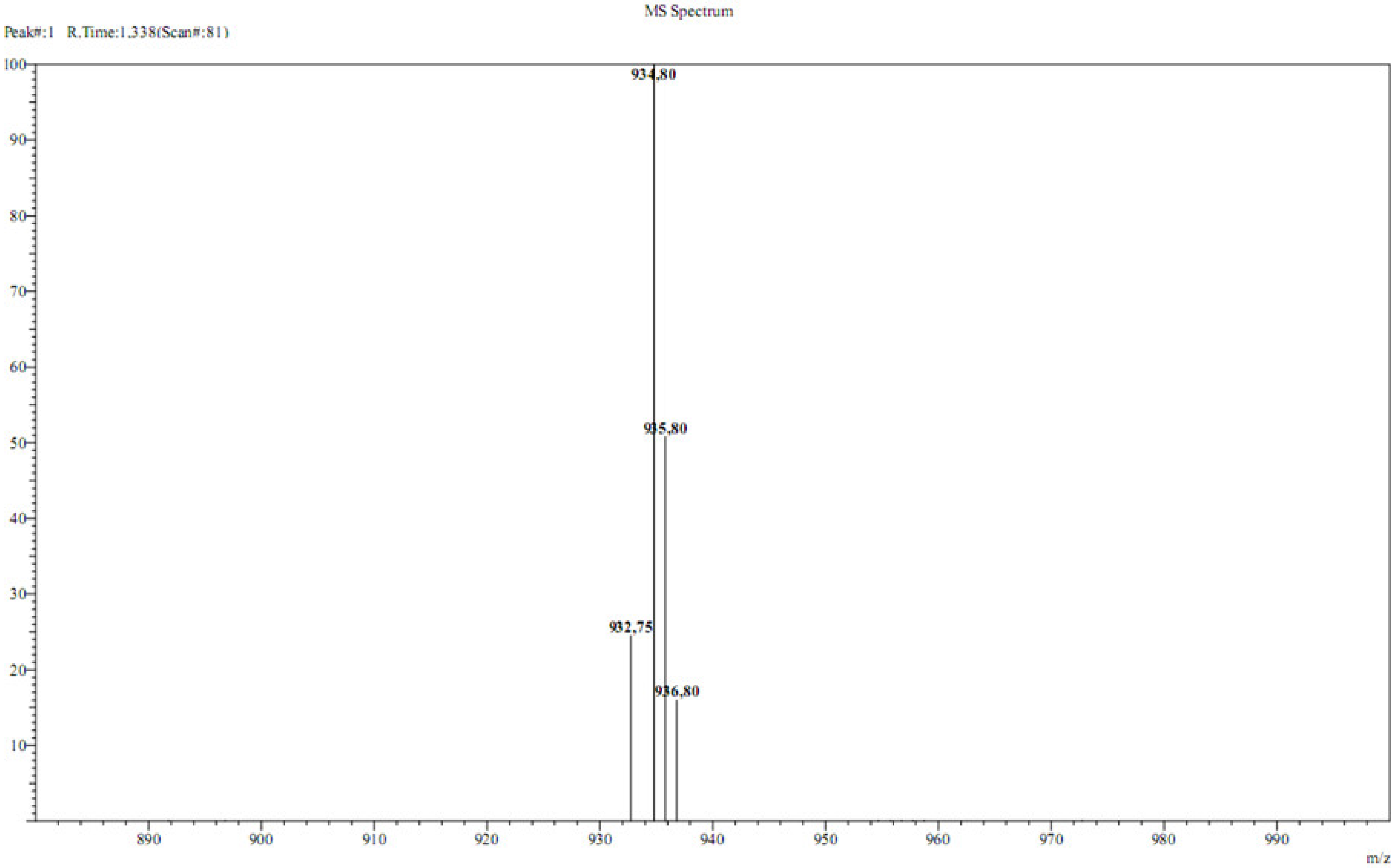
MS Spectrum
Peak#:1 R.Time:1.338(Scan#:81)
934,80
935,80
932,75
936,80
m/z
